# Structure and encapsulation of carbonic anhydrase within the α-carboxysome

**DOI:** 10.1073/pnas.2523723122

**Published:** 2025-11-12

**Authors:** Pei Cing Ng, Oluwatobi Adegbite, Tianpei Li, Arnaud Baslé, Jon Marles-Wright, Lu-Ning Liu

**Affiliations:** ^a^Institute of Systems, Molecular and Integrative Biology, University of Liverpool, Liverpool L69 7ZB, United Kingdom; ^b^Biosciences Institute, Faculty of Medical Sciences, Newcastle University, Newcastle upon Tyne NE2 4HH, United Kingdom; ^c^Ministry of Education Key Laboratory of Evolution and Marine Biodiversity, Frontiers Science Center for Deep Ocean Multispheres and Earth System and College of Marine Life Sciences, Ocean University of China, Qingdao 266003, China

**Keywords:** carboxysome, carbonic anhydrase, carbon fixation, encapsulation, protein assembly

## Abstract

Carboxysomes in cyanobacteria and certain proteobacteria enable efficient CO_2_ fixation by encapsulating ribulose-1,5-bisphosphate carboxylase/oxygenase (Rubisco) and carbonic anhydrase (CA) within a semipermeable shell. Sequestered CA catalyze the rapid interconversion of CO_2_ and HCO_3_^−^, supplying elevated levels of CO_2_ to boost Rubisco carboxylation. Despite its essential role, the structure and encapsulation of CA within carboxysomes remain poorly understood. Here, we determined the molecular structure of α-carboxysomal CA from the model chemoautotrophic bacterium *Halothiobacillus neapolitanus* (*Hn*CsoSCA). *Hn*CsoSCA adopts a trimer-of-dimers oligomeric structure without the incorporation of a zinc ion at its symmetric center. Using synthetic minishells, we demonstrate that *Hn*CsoSCA interacts with the CsoS1A shell hexamer and is incorporated into the minishells at the inner surface, independent of the CsoS2 linker protein. *Hn*CsoSCA truncations suggest nonspecific interactions between *Hn*CsoSCA and CsoS1A. We further show that *Hn*CsoSCA bridges Rubisco and the shell facets. Our study offers insights into the assembly and encapsulation mechanisms of α-carboxysomes and provides the framework for reprogramming carboxysome structures for synthetic biology and biotechnological applications.

## Significance

Carbonic anhydrase plays a pivotal role in biological carbon capture by catalyzing the interconversion of CO_2_ and bicarbonate. This study provides insights into the structure and encapsulation of carbonic anhydrase (CA) within the α-carboxysome, a proteinaceous organelle for CO_2_ fixation. The results reveal that α-carboxysomal CA from the chemoautotrophic, nonphotosynthetic bacterium *Halothiobacillus neapolitanus* (*Hn*CsoSCA) forms a trimer-of-dimers structure in the absence of a zinc ion at the hexameric interface. Using synthetic α-carboxysome shells, we show that *Hn*CsoSCA interacts with the CsoS1A shell hexamer and bridges the shell facet and Rubisco. These findings advance our understanding of α-carboxysome assembly and encapsulation mechanisms, paving the way for potential applications of carboxysome structures in synthetic biology and biotechnology.

Biological carbon fixation is a fundamental process in nature that enables organisms to convert inorganic carbon dioxide into organic compounds, forming the basis of global carbon cycles and sustaining life on Earth. This process is primarily carried out by autotrophic organisms, including plants, algae, and certain bacteria, through various metabolic pathways, with the Calvin–Benson–Bassham cycle being the most prevalent. In recent years, there has been growing interest in harnessing and enhancing biological carbon fixation mechanisms to address environmental challenges and develop sustainable biotechnological applications (1).

Carboxysomes are specialized proteinaceous organelles that play a central role in carbon fixation, as part of the CO_2_-concentrating mechanisms in cyanobacteria, some proteobacteria, and actinobacteria (2–5). The carboxysome comprises a polyhedral shell that encapsulates ribulose-1,5-bisphosphate carboxylase/oxygenase (Rubisco) and carbonic anhydrase (CA) (6–10). HCO_3_^−^ is accumulated intracellularly by membrane-bound transporters and CO_2_-uptake complexes and diffuses through the selectively permeable carboxysome shell into the interior (11); it is then rapidly converted to shell-impermeable CO_2_ by the encapsulated zinc metalloenzyme, CA. The architecture of the carboxysome enables an elevated steady-state concentration of CO_2_ around Rubisco, to levels that exceed the Michaelis constant (*K*_m_) of Rubisco, ensuring efficient carbon fixation and circumventing photorespiration.

Two convergent lineages of carboxysomes have been identified: the α- and β-types, which differ in the form of internal cargo, protein composition, and assembly mechanism (12–14). Of the eight classes of CA that have been identified (15), only β-, γ-, and ι-type CAs were found to be (nonexclusively) associated with carboxysomes. The β- and γ-type CAs have been identified in β-carboxysomes. The CcmM protein of β-carboxysomes has an N-terminal γ-class CA-like (γ-CAL) domain which is functional in *Thermosynechococcus elongatus* BP-1 (16) and *Nostoc* sp. PCC 7120 (17). However, in *Synechocystis* sp. PCC 6803 and *Synechococcus elongatus* PCC 7942, the CcmM γ-CAL domain has devolved into an inactive CA (18). CcaA, a β-type CA, is the functional CA in those species (19–23). Certain cyanobacterial species also contain *ccaA* and CcmM γ-CAL domains that are likely functional, and it is possible that CA activity is contributed by both types of CA in the β-carboxysomes of those species (16). In comparison, canonical α-carboxysomes utilize β-type CAs, namely CsoSCA (24). In *Thiomicrospira* strains where *csoSCA* is absent in the *cso* locus of the α-carboxysome, recent evidence suggests that ι-type CAs might be the functional α-carboxysomal CA instead (25). Deletion of CAs in α- and β-carboxysomes causes a high CO_2_-requiring phenotype, indicating their essential roles in carboxysome function (8, 19, 21, 23, 26).

The oligomeric states of β-carboxysomal CAs and their encapsulation have been well studied. It was previously shown that homotrimers of the CcmM γ-CAL domains interact with both CcaA and Rubisco, forming a precarboxysome condensate (27). Homotrimeric γ-CAL are also capable of associating together in a head-to-head fashion, which increases local protein concentration in the precarboxysome condensate and induces further β-carboxysome biogenesis. A crystal structure of *Synechocystis* sp. PCC 6803 CcaA revealed a hexameric form of the enzyme (28). For the α-carboxysome, early studies suggested that CsoSCA from the chemoautotrophic bacterium *Halothiobacillus neapolitanus* (*H. neapolitanus*) functions as a dimeric form with a tight association to the α-carboxysome shell (6, 29, 30). This was consistent with another finding demonstrating the presence of CsoSCA within synthetic empty α-carboxysome shells (31). Absolute quantification further revealed that ~120 copies of CsoSCA are sequestered within the *H. neapolitanus* α-carboxysome, which appeared to be sufficient to support up to 450 Rubisco holoenzymes (32). However, recent cryoelectron microscopy (cryo-EM) and cryoelectron tomography studies on the structures of α-carboxysomes from various organisms, including *H. neapolitanus* (33–35)*, Cyanobium* sp. PCC 7001 (34, 36) and *Prochlorococcus* MED4 (37), failed to determine the presence, localization, and in situ structure of CsoSCA within α-carboxysomes. Interestingly, a recently determined crystal structure of CsoSCA from *Cyanobium* (*Cy*CsoSCA) showed that CsoSCA exists as a homohexameric structure mediated by a zinc ion at the symmetric center (38). In vitro reconstitution and cryo-EM analysis suggested that Rubisco binds to the N-terminal peptide of CsoSCA, leading to the hypothesis that CsoSCA is recruited to the α-carboxysome by Rubisco (39). However, the exact oligomeric structure of CsoSCA and how it is encapsulated within the α-carboxysome remain elusive.

Here, we determined the structure of CsoSCA from *H. neapolitanus* (*Hn*CsoSCA) at 2.51 Å resolution using single-particle cryo-EM. Like *Cy*CsoSCA, *Hn*CsoSCA exhibited a trimer-of-dimers organization; unlike *Cy*CsoSCA, the *Hn*CsoSCA hexamer lacks the requirement for a zinc ion at its symmetric center, utilizing hydrophobic interactions instead. We further dissected the encapsulation mechanisms of *Hn*CsoSCA by using synthetic minimal α-carboxysome shells. Our results revealed that CsoSCA interacts with CsoS1A and can be incorporated into the minishells at the inner surface, independent of the linker protein CsoS2. We also provide evidence that CsoSCA bridges Rubisco and the shell facets. Our study provides insights into the assembly and encapsulation mechanisms of α-carboxysomes and opens further possibilities for repurposing carboxysome structures for a variety of biotechnological and biomedical applications.

## Results

### Structure of the *Hn*CsoSCA Hexamer.

To determine the structure of *Hn*CsoSCA, we expressed *Hn*CsoSCA fused with a N-terminal Twin-Strep tag and the B1 domain of Streptococcal Protein G (GB1) (40) using *Escherichia coli* BL21(DE3) (*SI Appendix*, Fig. S1*A*). Addition of the GB1 domain greatly improved CsoSCA solubility and stability (*SI Appendix*, Fig. S1 *B* and *C*). Following Strep-tag affinity chromatography, purified Twin-Strep-GB1-CsoSCA was eluted predominantly at approximately 420 kDa on size exclusion chromatography coupled with multiangle static light scattering (SEC-MALS; *SI Appendix*, Fig. S1*D*), indicating a hexameric state of *Hn*CsoSCA in solution, which is consistent with previous studies (38).

**Fig. 1.**
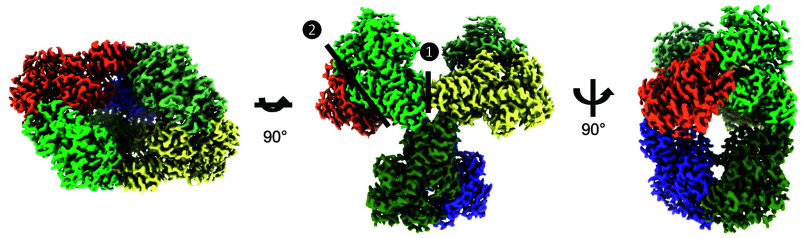
Architecture of the *Hn*CsoSCA hexamer. Single-particle cryo-EM derived map of the *H. neapolitanus* CsoSCA (*Hn*CsoSCA) hexamer shown as a solid surface. The density map is shown in top, front, and side views from left to right. Each protomer in the structure is given a distinct color. Interface 1 represents the hexamer interface whereas interface 2 is the dimer interface.

**Fig. 2.**
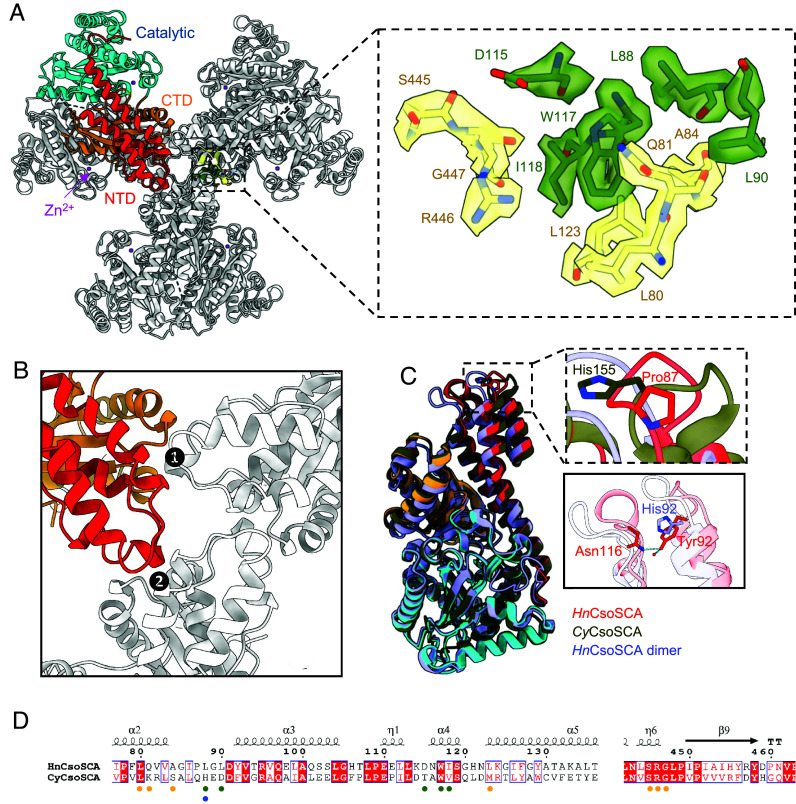
The hexamer interface of *Hn*CsoSCA. (*A*) Cartoon representation of *Hn*CsoSCA. The N-terminal domain (NTD; red), catalytic domain (cyan), and C-terminal domain (CTD; orange) of a single protomer are indicated. The catalytic zinc ion is shown as a magenta sphere. Residues involved in the hexamer interface are shown in the *Inset* as sticks, where residues from two distinct protomers are shown in different colors. The unresolved segment connecting the NTD and the catalytic domain is shown as dashed lines. (*B*) The two interdimeric interfaces of each *Hn*CsoSCA protomer that mediate oligomerization at the threefold symmetry axis. Domains of the protomer are highlighted according to the color scheme in *A*. (*C*) Superposition of the protomer from *Hn*CsoSCA (red; this work), hexameric CsoSCA from *Cyanobium* PCC 7001 (*Cy*CsoSCA; dark green; PDB: 8THM) and the *Hn*CsoSCA dimer (purple; PDB: 2FGY). The *Top Inset* shows His155 of *Cy*CsoSCA and the corresponding residue at that position in *Hn*CsoSCA, Pro87. The *Bottom Inset* shows Tyr92 of the *Hn*CsoSCA hexamer and His92 of the *Hn*CsoSCA dimer. The hydrogen bonding between Tyr92, a water molecule, and Asn116 of the *Hn*CsoSCA hexamer is shown in blue dotted lines. Important residues are shown as sticks and annotated. The water molecule is shown as a red sphere. (*D*) Sequence alignment of *Hn*CsoSCA and *Cy*CsoSCA. Residues involved in oligomerization shown in a are annotated with yellow and green circles. His155 of *Cy*CsoSCA is marked by a blue circle. Residues are numbered according to the *Hn*CsoSCA sequence and the secondary structure of *Hn*CsoSCA is shown above the alignment.

We then resolved the structure of *Hn*CsoSCA at 2.51 Å resolution using single-particle cryo-EM (Fig. 1 and *SI Appendix*, Fig. S2 and Table S1). The overall oligomeric state of *Hn*CsoSCA is a homohexamer, with D3 symmetry formed from a trimer-of-dimers, resembling the *Cy*CsoSCA homolog (38). The monomer of *Hn*CsoSCA showed good map densities for residues 48 to 514 of the native sequence with a short unresolved loop between residues 143 and 154 in the region connecting the N-terminal and catalytic domains. The catalytic domain of each monomer contains an α/β fold that is structurally characteristic of β-CAs, such as β-carboxysomal CcaA (28). Within the active site of *Hn*CsoSCA, we observed electron density indicative of metal ion coordination and assigned the density as a Zn^2+^ ion based on bond lengths and angles and biochemical features of this enzyme (Fig. 2*A*). The Zn^2+^ molecule is coordinated by Cys173, His242, and Cys253 in the catalytic domain of each monomer (*SI Appendix*, Fig. S2*E*), resulting in six zinc ions in the hexamer.

Closer structural analysis, and interface analysis using the PDBePISA tool, revealed that the dimerization interface of *Hn*CsoSCA is primarily stabilized by nonpolar contacts, burying ~3,000 Å^2^ of surface area. Hydrogen bonds and salt bridges between neighboring catalytic and C-terminal domains further stabilize the dimer (*SI Appendix*, Fig. S3). This is consistent with the X-ray crystal structure of dimeric *Hn*CsoSCA reported in previous studies (29). The higher-order oligomerization into trimers-of-dimers is formed by interactions between the terminal domains of the monomers to produce two equivalent trimerization interfaces related by the D3 symmetry of the oligomer. These trimerization interfaces are primarily hydrophobic in nature and are smaller than the dimerization interface at ~800 Å^2^ (Fig. 2*A*). These interfaces comprise a small cleft formed between α-helical residues on the N- and C-terminal domains of one CsoSCA protomer, which allows contacts with residues in the N-terminal loop regions of the protomer in the adjacent dimer (Fig. 2*B*). Each protomer interacts with two adjoining monomers in this fashion, providing a cleft site on one end and reaching through with its N-terminal loops to associate with another monomer at the other end. All domains of *Hn*CsoSCA are important for hexamer formation, as *Hn*CsoSCA loses its hexameric state when expressed in differently truncated forms (*SI Appendix*, Fig. S4). Furthermore, the importance of the C-terminal domain in hexamer formation explains why it has been evolutionarily conserved in the enzyme, even though it does not contribute to the catalytic function (29). Residues identified to mediate hexamer formation are broadly conserved among α-carboxysomal CsoSCA (Fig. 2*A* and *SI Appendix*, Fig. S5*A*).

The monomer of the *Hn*CsoSCA hexamer superimposes well with those of the *Cy*CsoSCA hexamer (PDB: 8THM) and *Hn*CsoSCA dimer (PDB: 2FGY), with RMSD values of 0.882 Å and 1.277 Å, respectively (Fig. 2*C*). Oligomerization of the *Cy*CsoSCA hexamer was proposed to be driven by coordination of a zinc ion between three interdimeric His155 residues in an octahedral (His)_3_(H_2_O)_3_ coordination sphere (38). His155 of *Cy*CsoSCA resides in the N-terminal domain “hook motif,” a feature that distinguishes CsoSCA from CsoSCA homologues residing outside of the α-carboxysome *cso* operons (38, 41). While this motif is present in our *Hn*CsoSCA structure and forms part of the hexameric interface, *Hn*CsoSCA has Pro87 instead of His155 (Fig. 2*C*), precluding metal ion coordination. Additionally, there is poor sequence conservation of the α2-α3 loop where *Hn*CsoSCA Pro87 and *Cy*CsoSCA His155 are located (Fig. 2*D*), and the conformations of the loop in both structures are markedly different (Fig. 2*C*). Indeed, there is a lack of density corresponding to a zinc ion and its coordination sphere at the apex of the *Hn*CsoSCA hexamer, where a structural zinc ion was clearly identified in *Cy*CsoSCA (*SI Appendix*, Fig. S5*B*). This discrepancy suggests a structural variation between the two CsoSCA proteins and indicates that metal ion-mediated oligomerization is not highly conserved among CsoSCAs. Intriguingly, the residues responsible for *Hn*CsoSCA hexamerization, which are strongly conserved (L80, W117, S445, R446, and G447) (*SI Appendix*, Fig. S5*A*), are also present in *Cy*CsoSCA (Fig. 2*D*). Structural analysis of *Cy*CsoSCA further revealed that the corresponding residues (L147, W184, R506, G507) are also involved in hydrophobic interactions and hydrogen bonding at the hexamer interface (*SI Appendix*, Fig. S5*C*), although His155 is still the primary element responsible for maintaining the stability of the *Cy*CsoSCA hexamer structure.

While His155 of *Cy*CsoSCA is not preserved in *Hn*CsoSCA, histidine is the most common amino acid at this position across α-carboxysomal CsoSCA sequences (*SI Appendix*, Fig. S5*A*). Phylogenetic analysis of CsoSCA from typical α-carboxysome loci (41) shows that CsoSCA variants with and without the histidine residue evolved concurrently (*SI Appendix*, Fig. S6). The presence or absence of this histidine in CsoSCA appears to be lineage-specific. Sequence alignment reveals that the histidine residue is highly conserved in the cyanobacterial clade, to which *Cy*CsoSCA belongs, with ~91% of cyanobacterial *csoSCA* genes encoding a conserved histidine. In contrast, other bacterial classes show a more balanced distribution, with only ~49% of CsoSCA sequences containing the conserved histidine. These findings suggest that hydrophobic interactions are likely the primary force driving the oligomerization of α-carboxysomal CsoSCA proteins. Additionally, it appears that a subset of CsoSCA has evolved to use metal coordination as an additional mechanism to enhance the stability of the hexamer interface.

Notably, the *Hn*CsoSCA dimer reported previously contained an inadvertent point mutation of Tyr92 to His92 (29). In the *Hn*CsoSCA hexamer, Tyr92 forms hydrogen bonds with a water molecule and the carboxyamide side chain of Asn116 (Fig. 2*C*). Mutation of Tyr92 to His92 would weaken or abolish the hydrogen bonding and likely destabilize the hexameric interface. This would explain why *Hn*CsoSCA dimers were observed initially, instead of hexamers.

### Nonspecific Interactions with Shell Proteins Mediate CsoSCA Recruitment.

**Fig. 3.**
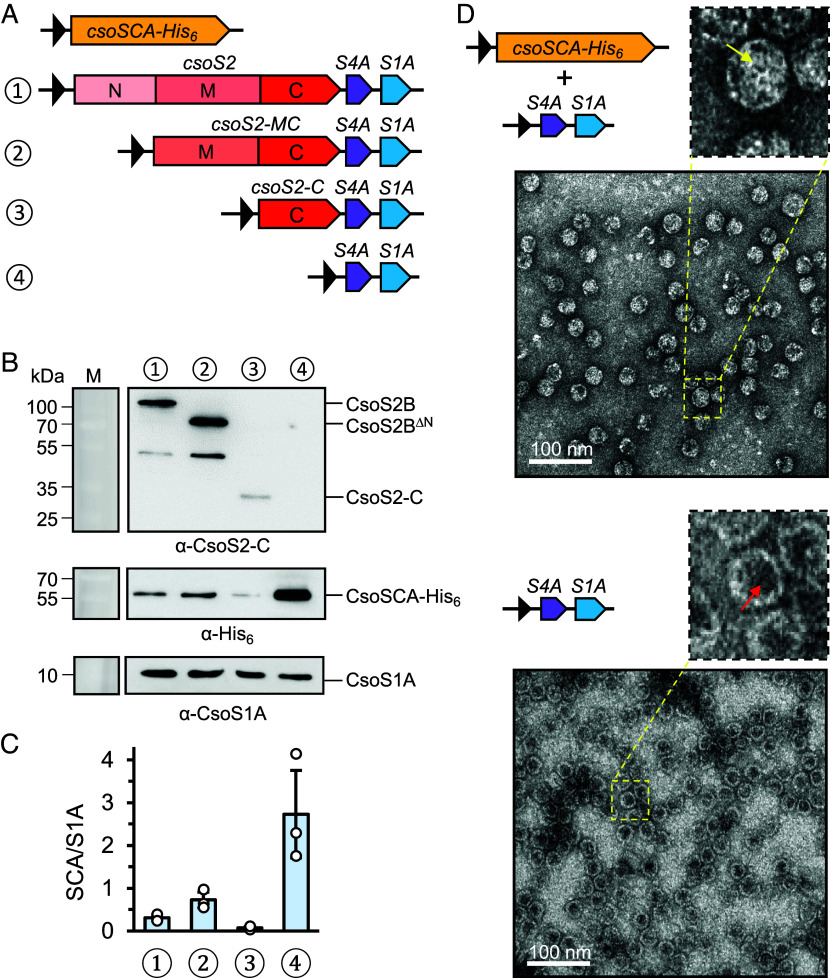
CsoSCA assembly in the minishell is independent of CsoS2. (*A*) Schematic diagram of the experimental set-up. CsoSCA with a C-terminal 6xHis tag was independently coexpressed with four different minishell constructs for purification. Construct 1 contains full-length *csoS2*, construct 2 contains *csoS2* truncated at the N-terminal domain, construct 3 contains *csoS2* truncated at the N-terminal domain and middle region, leaving only the C-terminal domain, and construct 4 does not contain any *csoS2*. (*B*) Western blotting of purified minishells from the experiment described in a. Samples were normalized to the same amount of CsoS1A proteins detected by α-CsoS1A. Purifications were repeated three times (*n* = 3), and a representative Western blot is shown. (*C*) Relative quantification of copurified CsoSCA from the experiment described in a, normalized by amount of CsoS1A (*n* = 3). Quantification was performed with Western blotting experiments shown in *B*. Individual data points are shown. Error bars represent the SD. (*D*) Negative-staining transmission electron micrographs of purified minishells from coexpression of minishell construct 4 with the *csoSCA* construct (*Top*), or minishells purified from expression of minishell construct 4 alone (*Bottom*). *Insets* show the different internal densities between the two micrographs indicated by the yellow and red arrows. Scale bar represents 100 nm.

Previous immunoblot and mass spectrometry data revealed the presence of *Hn*CsoSCA (hereafter CsoSCA) in *H. neapolitanus* α-carboxysome shells that lack Rubisco, suggesting that encapsulation of CsoSCA within the α-carboxysome relies on shell or shell-associated proteins (30, 31). Additionally, β-carboxysomal CcaA was shown to form a precarboxysome condensate through interactions with CcmM (27). To test whether the shell or shell-associated proteins mediate CsoSCA encapsulation, we created a series of minishells, either lacking the linker protein or containing truncated linker proteins, as caging systems to dissect CsoSCA encapsulation. In this minishell system, heterologous expression of CsoS2 (linker protein), CsoS1A (the major shell hexamer), and CsoS4A (shell pentamer) from *H. neapolitanus* resulted in the assembly of synthetic icosahedral shells ranging from 25 to 40 nm (42). CsoS2 was systematically truncated from the N terminus, generating two additional minishell constructs: *csoS2-MC*, which has an N-terminal CsoS2 truncation, and *csoS2-C*, which lacks both the CsoS2 N-terminal and middle regions (Fig. 3*A*). CsoSCA-His_6_ (CsoSCA fused to a C-terminal His_6_ tag for immunoblot detection) was coexpressed with each of these constructs, as well as a minishell construct with full-length CsoS2 as a control, followed by purification of minishells as reported previously (42). We found that the amount of copurified CsoSCA was at least four-fold higher in minishells lacking CsoS2 (Fig. 3 *B* and *C*). Hereafter, “minishells” specifically refers to the CsoS4A-CsoS1A minishells generated from Construct 4 (S4A-S1A) in Fig. 3*A*. Furthermore, negative-staining transmission electron microscopy (TEM) revealed a different internal density pattern of the minishells coexpressed with CsoSCA compared to empty minishells (Fig. 3*D*), suggesting successful recruitment of CsoSCA within the minishells.

When expressed by itself, CsoSCA is not detected in the corresponding minishell elution fraction, having eluted earlier in the purification (*SI Appendix*, Fig. S7). When CsoSCA was coexpressed with the minishell, free CsoSCA was detected in the same fraction that individual CsoSCA enzymes eluted (*SI Appendix*, Fig. S7 *B* and *C*), as well as in the minishell elution fractions (*SI Appendix*, Fig. S7*D*), confirming that CsoSCA detected in the latter fractions were encapsulated in minishells. Intriguingly, CsoSCA with an N-terminal Strep-tag II (StrepII-CsoSCA) pulls down CsoS1A when coexpressed with the minishell (*SI Appendix*, Fig. S8 *A* and *B*). It is assumed that the detected CsoS1A exists as nascent shells, which is unsurprising given that the constructs are being overexpressed, with no regulatory system in place to ensure only fully assembled minishells exist in the cell. We were unable to verify the absence or presence of CsoS4A in the elution of CsoSCA in the pull-down assay. The flow-through of this in vivo pull-down assay was further purified using the minishell purification protocol. As expected, CsoSCA was detected in the elution of fully assembled minishells (*SI Appendix*, Fig. S8*C*), which exhibited a negative-staining TEM staining pattern consistent with that shown before (Fig. 3*D* and *SI Appendix*, Fig. S8*D*). Collectively, our data indicate an interaction between CsoSCA and CsoS1A, and that CsoSCA can be incorporated into minishells composed of CsoS4A and CsoS1A, without the assistance of the linker protein CsoS2. This finding is in agreement with earlier reports that failed to detect interactions between CsoSCA and CsoS2 (39).

**Fig. 4.**
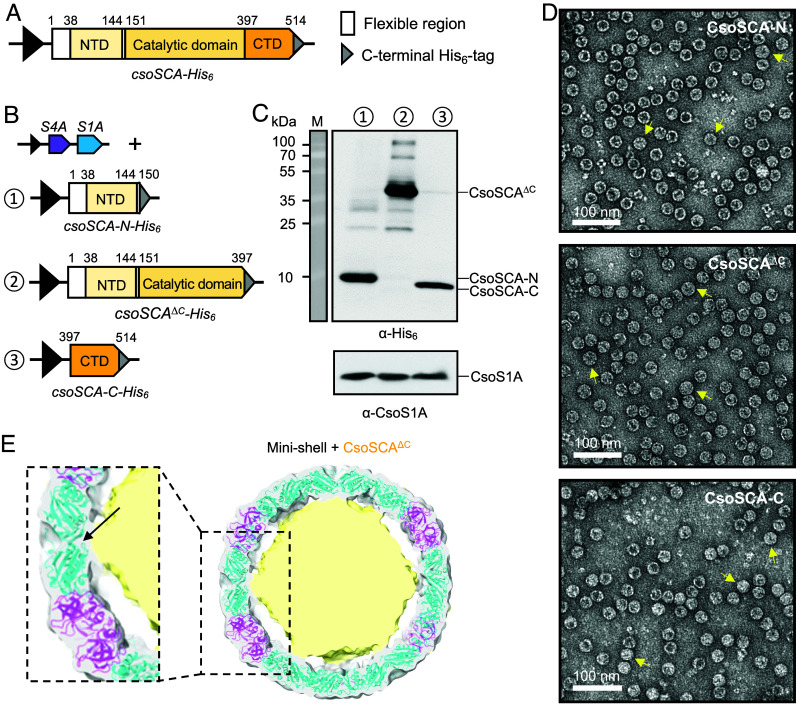
Interactions of the CsoSCA domains with the minishell. (*A*) Schematic representation of the primary structure of *H. neapolitanus* CsoSCA. Residue numbering is shown. (*B*) Schematic diagram of the experimental set-up. The minishell construct is individually coexpressed with three truncated *csoSCA* variants. 1 represents the N-terminal domain of CsoSCA *(csoSCA-N)*, 2 represents CsoSCA with a C-terminal domain truncation *(csoSCA^ΔC^)*, 3 represents the C-terminal domain of CsoSCA *(csoSCA-C)*. (*C*) Western blotting of purified minishells in the experiment described on the *Left*. Samples were normalized to the amount of CsoS1A proteins detected by α-CsoS1A. (*D*) Negative-staining transmission electron micrographs of purified minishells from the purifications described in *B*. Scale bar represents 100 nm. The yellow arrows indicate minishells with internal density. (*E*) Symmetry relaxed cryo-EM density map of purified minishell/CsoSCA^∆C^. The map is shown at low contouring and fitted with the *T* = 4 minishell model determined previously (PDB: 8B11). The map was processed in ChimeraX using a Gaussian filter with a SD of two sigma and displayed at a contour level of 0.025. The map and atomic model are clipped at the front and back to allow clear visualization of the internal density. The minishell model is depicted as a cartoon (cyan for CsoS1A, magenta for CsoS4A) and the internal density on the map is colored in light yellow. (*Inset*) The arrow indicates the connection between the internal density and the central pore of a CsoS1A hexamer. Conversely, there is a lack of contact between the inner density and CsoS4A.

We then investigated the interactions of CsoSCA with minishell proteins, by determining the specific regions of contact between CsoSCA and shell hexamers/pentamers. Cryo-EM of purified minishell/CsoSCA hinted at the presence of internal density in two-dimensional (2D) classes, but density corresponding to CsoSCA was not identified in the reconstructed three-dimensional (3D) maps, suggesting high disorder of the internal density. Following this, we sought to identify interacting regions by truncating CsoSCA. CsoSCA can be structurally divided into three domains: an N-terminal domain (NTD; residues 38 to 144), a catalytic domain (residues 151 to 397), and a C-terminal domain (CTD; residues 397 to 514) (Figs. 2*A* and 4*A*). In addition, there is a flexible region (residues 1 to 37) that precedes the NTD and a flexible linker region (residues 145 to 150) connecting the NTD and catalytic domain (Fig. 4*A*). We generated constructs that express either the NTD or CTD of CsoSCA, as well as a CTD deletion of CsoSCA; all three constructs contain a His_6_ tag fused at the C-terminus of CsoSCA for immunoblot detection (Fig. 4*B*). The CsoS4A-CsoS1A minishell construct was then coexpressed with each of the individual CsoSCA mutant constructs, which yielded soluble proteins (*SI Appendix*, Fig. S9*A*). SEC-MALS of individually expressed and purified mutant CsoSCA constructs further confirmed that the expressed proteins were soluble, although a fraction of the NTD of CsoSCA is prone to aggregation and forms multimers rather than monomers when expressed by itself (*SI Appendix*, Fig. S4). Immunoblot analysis showed that all the CsoSCA mutants were incorporated within minishells when coexpressed and purified (Fig. 4*C*). Negative-staining TEM revealed that the internal densities of minishells from these three purifications were consistent with those observed for the minishell/CsoSCA sample (Figs. 3*D* and 4*D*). Together, these results suggest that the NTDs and CTDs of CsoSCA can independently interact with the minishell to mediate CsoSCA encapsulation.

To investigate the molecular basis for these interactions, we evaluated the CsoSCA–shell association by performing single-particle cryo-EM on purified minishell/CsoSCA^ΔC^ and minishell/CsoSCA-C. Initial analysis of micrographs from a preliminary dataset revealed the presence of *T* = 3 and *T* = 4 shells in both samples (*SI Appendix*, Fig. S9*B*), which is consistent with the shell population observed from expression of empty minishells (42). Notably, we observed a marked increase in the internal density in *T* = 4 shells compared to *T* = 3 at lowered contour levels. Therefore, we specifically purified *T* = 4 minishells by collecting elution fractions from ion exchange chromatography and screening for fractions enriched with larger minishells by negative-staining TEM (*SI Appendix*, Fig. S9*C*). The cryo-EM density maps of *T* = 4 shells from both CsoSCA^ΔC^ (2.36 Å) and CsoSCA-C (3.05 Å) minishells reveal a well-defined shell density, to which the previously published *T* = 4 empty minishell structure (PDB: 8B11) (42) was fitted and refined (Fig. 4*E* and *SI Appendix*, Figs. S10–S12 and Table S1). To better visualize any internal density, we employed a series of data processing procedures to enhance structural features within the shell, including particle subtraction to remove shell densities, masked 3D classification, refinement with no imposed symmetry, symmetry relaxation, and nonuniform refinement. Despite these efforts, we were unable to resolve ordered internal densities in our cryo-EM maps. In contrast to the previously reported CsoS2-containing minishells (42), we found no evidence of structured peptide interactions between CsoSCA and the internal shell surface. This observation suggests that CsoSCA interacts with the α-carboxysome shell in a nonspecific and dynamic manner. Closer inspection showed that the disordered internal density in both maps contacts the CsoS1A hexamer but not the CsoS4A pentamer (Fig. 4*E* and *SI Appendix*, Fig. S12). This supports our earlier findings (*SI Appendix*, Fig. S8) and suggests that CsoSCA is likely localized to the α-carboxysome shell facet rather than the shell vertices. The minishell/CsoSCA-C map has less internal density than the minishell/CsoSCA^ΔC^ map, consistent with the smaller molecular mass of CsoSCA-C relative to CsoSCA^ΔC^ (Fig. 4*C* and *SI Appendix*, Fig. S4). In addition, the internal density in both maps appear to contact CsoS1A hexamers in proximity to their central pore regions, although this remains to be further validated in future studies.

### CsoSCA–Shell Interactions in the Context of Rubisco.

The interactions between CsoSCA and shell proteins, and the binding of the flexible N-terminal peptide of CsoSCA to Rubisco as suggested by recent studies (39), led to the hypothesis that CsoSCA may contribute to recruitment of Rubisco. To verify this, we coexpressed minishells (lacking CsoS2) with both CsoSCA and Rubisco (*SI Appendix*, Fig. S13*A*). The results showed that minishells containing CsoSCA exhibited a higher content of encapsulated CbbL (the large subunit of Rubisco), compared to minishells lacking CsoSCA (*SI Appendix*, Fig. S13*B*). The limited interior space of the minishell suggests that it is more likely that individual CbbL subunits, rather than the fully formed CbbL_8_S_8_ enzyme, is being incorporated by CsoSCA into the minishell. The limited content of CbbL recruited by CsoSCA and CsoSCA-N may be attributed to spatial constraints imposed by the minishell structures (*SI Appendix*, Fig. S9). Indeed, recent studies have shown that expansion of the α-carboxysome shell requires the middle or C-terminal region of CsoS2 and other shell proteins (42–45).

To corroborate our findings, we coexpressed StrepII-CsoSCA with the minishell and Rubisco (*SI Appendix*, Fig. S13*A*), utilizing StrepII-CsoSCA as the bait protein for an in vivo pull-down assay as shown before (*SI Appendix*, Fig. S8). A C-terminal His_6_ tag was fused to CbbS (CbbS-His_6_, the small subunit of Rubisco) for identification. The results show that StrepII-CsoSCA was able to simultaneously pull down CbbL, CbbS-His_6_, and CsoS1A (*SI Appendix*, Fig. S13*C*). Altogether, our results provide strong evidence for the interaction between CsoSCA and Rubisco. The findings also suggest that CsoSCA–Rubisco interactions may contribute to recruitment of Rubisco into α-carboxysome shells, although CsoS2-mediated Rubisco encapsulation appears to be dominant (34, 46).

To further validate our observations from the minishell platform, we employed heterologous expression of full α-carboxysome shells to create an environment more akin to the native system (31). We expressed and purified two types of structures: intact α-carboxysomes that were expressed from the entire *cso* operon including *csoS1D*, and α-carboxysome shells that lack Rubisco (*SI Appendix*, Fig. S14*A*). Purified α-carboxysomes and empty α-carboxysome shells exhibit polyhedral morphology (*SI Appendix*, Fig. S14*B*), with diameters of 135.0 ± 12.3 nm (*n =* 104) and 115.7 ± 16.1 nm (*n =* 77) respectively, in agreement with previous observations (47, 48). We detected CsoSCA in purified α-shells despite the lack of Rubisco (*SI Appendix*, Fig. S14*C*). This result corroborates earlier findings (31) and confirms that CsoSCA can be encapsulated within the α-carboxysome through direct interactions with the shell, independent of Rubisco. However, the amount of CsoSCA in empty α-carboxysome shells was reduced compared to intact α-carboxysomes, suggesting that Rubisco stabilizes CsoSCA–shell interactions when bound to CsoSCA. It also implies that some CsoSCA proteins are incorporated into the α-carboxysome through independent interactions with Rubisco, without assistance from the shell proteins.

## Discussion

Both Rubisco and CA are sequestered and functionally coordinated with each other within the carboxysome. CA catalyzes rapid interconversion of CO_2_ and HCO_3_^−^, effectively increasing the availability of inorganic carbon for fixation by Rubisco. It is particularly important in aquatic environments and for organisms living in alkaline conditions (49–51), where it helps maintain a sufficient CO_2_ supply for carbon fixation despite potential limitations in CO_2_ diffusion or availability. Despite its essential role in carboxysome physiology, the oligomeric structure and in situ organization of CA in the carboxysome are poorly understood. In this study, we determined the homohexameric structure of CsoSCA from *H. neapolitanus* and investigated how it is recruited into α-carboxysome shells composed of CsoS1A and CsoS4A shell proteins, as well as CsoSCA encapsulation in the context of Rubisco. Our study provides insights into the evolutionary variation of CA enzymes and α-carboxysome assembly. Additionally, these findings underscore the complex interplay between carboxysome components, which is fundamental for α-carboxysome formation and functionality.

### Carboxysomal β-CAs Assemble into Hexamers.

Both *Hn*CsoSCA and *Cy*CsoSCA belong to the β-class of CAs, which are widely distributed across archaea, bacteria, and eukaryotes (52). These enzymes exhibit diverse quaternary structures, ranging from dimers (the basic structural unit) (53–55) to more complex assemblies such as tetramers (56, 57), hexamers (28, 38), and octamers (58). Notably, the hexameric state has only been observed for carboxysomal β-CAs to date, including α-carboxysome CsoSCA from the chemoautotroph *H. neapolitanus* (Figs. 1 and 2) and the α-cyanobacterium *Cyanobium* (38), as well as β-carboxysome CcaA from the β-cyanobacterium *Synechocystis* sp. PCC 6803 (28). All three carboxysomal CAs assemble into a trimer-of-dimers conformation with D3 symmetry, although their size, orientation of dimers within the hexamer, and the hexamer interface differs between α- and β-carboxysomal CAs (*SI Appendix*, Fig. S15). The mode of hexamer formation appears to vary among β-CAs of α-carboxysomes. Some CsoSCA, such as *Hn*CsoSCA, rely only on hydrophobic interactions to drive hexamer formation (Fig. 2), whereas other clades of CsoSCA, especially cyanobacterial CsoSCA, contain a conserved histidine residue that likely enables coordination of structural zinc ions to stabilize the hexamer interface as seen in *Cy*CsoSCA (*SI Appendix*, Figs. S5 and S6). Other residues commonly found in protein zinc coordination spheres include cysteine, aspartate, and/or glutamate (59). For example, in the CsoSCA of *Sulfurivirga caldicuralii* DSM 17737 and *Thiohalospira halophila* HL3, aspartate/glutamate were found to replace the histidine residue. In addition, in a few γ-proteobacteria that are close relatives to the cyanobacterial clade, such as *Ectothiorhodospira marina* DSM 241 and *Rhabdochromatium marinum* DSM 5261, CsoSCA contains a cysteine residue instead of the conserved histidine. Further investigation into these structures could provide insights into the presence or absence of structural zinc ions in these CsoSCA and the diverse strategies employed by carboxysomal β-CAs for hexamer stabilization and assembly.

**Fig. 5.**
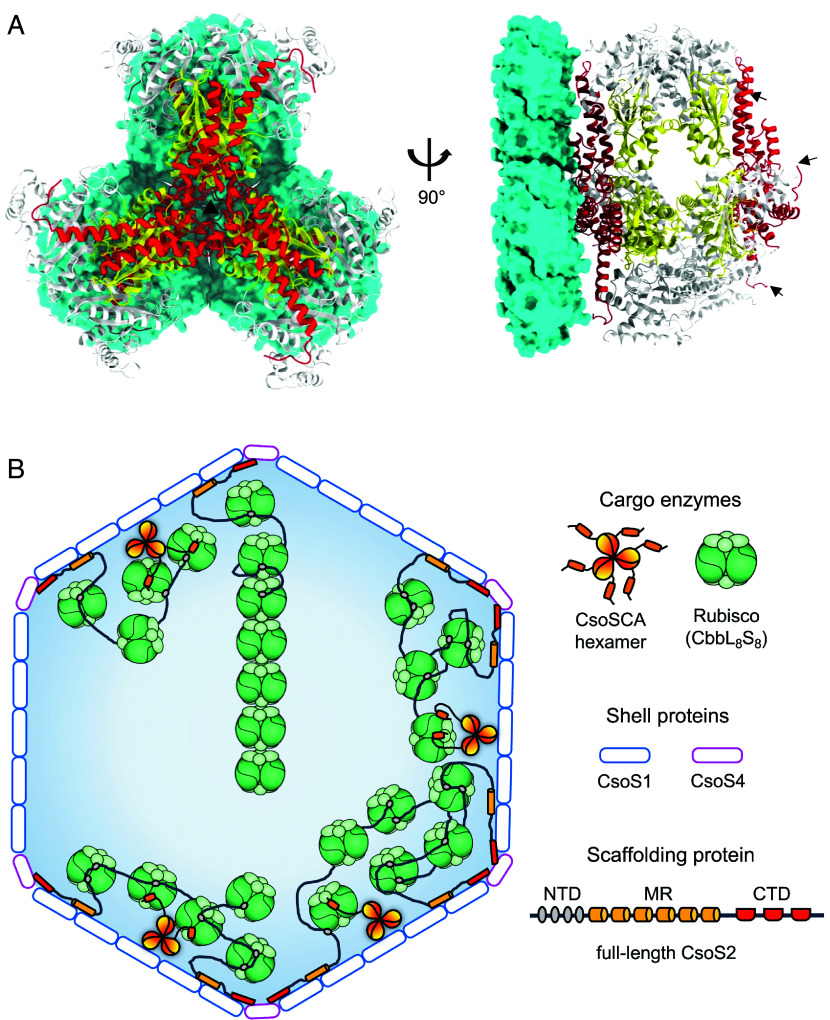
A hypothetic model of CsoSCA binding and its location in the α-carboxysome. (*A*) A proposed binding model of CsoSCA to the shell facets by superposition of their threefold axes, shown in front (*Left*) and side (*Right*) view. Three CsoS1A hexamers (PDB: 2EWH, cyan) depicted as a surface represent the α-carboxysome shell facet; generated through fitting of three 2EWH hexamers to the threefold axes of the *Prochlorococcus* α-carboxysome shell (EMD-37903). *Hn*CsoSCA (PDB: 9G4T) is shown as a cartoon, where red, white, and yellow delineates its NTD, catalytic domain, and CTD respectively. Front view: the black triangle specifies the threefold axes of *Hn*CsoSCA and the three CsoS1A hexamers. Side view: one side of *Hn*CsoSCA faces the α-carboxysome shell, and the other side faces the shell interior. The NTD of *Hn*CsoSCA is in close proximity to the shell facet. Black arrows denote disordered N-terminal regions that are free to bind to Rubisco. (*B*) An updated model of the α-carboxysome ultrastructure. CsoSCA binds to the shell facets (blue). The trimer-of-dimers structure of CsoSCA allows simultaneous binding to the shell and Rubisco. Each CsoSCA protomer has a flexible N-terminal tail, but only those interacting with Rubisco are shown for clarity. CsoS2 facilitates encapsulation of Rubisco via interactions at its N-terminal domain (NTD), and stitches the shell proteins together at its middle region (MR) and C-terminal domain (CTD). Two modes of Rubisco organization in the α-carboxysome are shown in the *Top* and *Bottom* of the schematic. For clarity, not all MR and CTD motifs of CsoS2 are shown, and only representative Rubisco molecules are drawn.

Why this specific oligomerization symmetry of β-CAs is only found for carboxysomal CAs remains unclear. Cooperative ligand binding, a common trait observed for protein oligomers, as exemplified by hemoglobin (60), has not been demonstrated for CsoSCA. Additionally, although *Cy*CsoSCA was found to be allosterically activated by RuBP, *Hn*CsoSCA is constitutively active (38). Notably, there is increasing evidence demonstrating the importance of multivalent interactions in protein condensate formation and carboxysome assembly. In the β-carboxysome, a single CcaA oligomer forms multiple interactions with its interacting partner CcmM (27). Our study suggests that CsoSCA binds both Rubisco and α-carboxysome shell proteins at distinct sites on an oligomer. Unlike β-carboxysomes, where there is no need for a symmetry match between CcaA and CcmM (28), we propose that CsoSCA binds to the α-carboxysome shell by overlapping its threefold axis with the threefold axis formed between three CsoS1 hexamers (Fig. 5*A*), which is further discussed below. This binding orientation, where CsoSCA binds to the shell facet through three protomers in the hexamer, ensures that the remaining three protomers are exposed to the shell interior for interactions with Rubisco. Therefore, it is likely that the hexameric form of carboxysomal β-CA exists to maximize the number of binding interactions and catalytic output, while maintaining high packing efficiency within the carboxysome.

### A Putative Model of CsoSCA Binding to the α-carboxysome Shell.

Our study shows that the interactions between CsoSCA and the shell appear to be nonspecific, with no defined peptide binding to the shell (Fig. 4 and *SI Appendix*, Fig. S12). This might be due to the lack of specific binding interfaces formed by multiple different shell proteins, or hetero-oligomers formed by shell paralogs in the minishell structures (45). We hypothesize that a more complete assembly of the α-carboxysome shell, with its full set of shell proteins incorporated at the appropriate curvature, may enhance the association of CsoSCA with the shell structure. This notion is somewhat supported by our findings of greater internal density appearing in the *T* = 4 minishells compared to the *T* = 3 minishells. We likewise did not identify internal density corresponding to CsoSCA from cryo-EM of purified minishells/full-length CsoSCA, despite evidence showing recruitment of CsoSCA in the minishells (Fig. 3 and *SI Appendix*, Fig. S7), likely through interactions between CsoSCA and CsoS1A (*SI Appendix*, Figs. S8 and S13). This suggests that the CsoSCA–CsoS1A interaction is perturbed during minishell assembly, presumably due to the limited interior space of the minishells.

Based on our results, we propose a speculative binding model of CsoSCA to the α-carboxysome shell facet (Fig. 5*A*). The CsoSCA hexamer likely binds to three CsoS1 hexamers on their convex surface by aligning its three-fold symmetry axis with the three-fold axis formed by three CsoS1 hexamers, in a dynamic fashion. In this orientation, one side of the CsoSCA hexamer lies flat against the shell facet, burying a substantial amount of surface area. It also places the NTD of CsoSCA close to the shell, facilitating binding interactions which could be electrostatic (*SI Appendix*, Fig. S16). Furthermore, sequence alignments reveal insertions on the NTD of CsoSCA that are only conserved in *csoSCA* associated with *cso* operons (38, 41), which suggests a carboxysome-specific function of the NTD. This corroborates our model of CsoSCA binding to the shell. It remains unclear whether the CTD of CsoSCA forms close contact with the shell. Careful inspection of the *Hn*CsoSCA structure reveals that the CTD is buried within the hexamer, with very little exposed surface area. Therefore, while individual CsoSCA-C monomers might be able to interact with and be encapsulated within the minishell (Fig. 4*C* and *SI Appendix*, Fig. S12), this interaction may not be biologically relevant in the native α-carboxysome. As the minishell system is not a complete representation of the native α-carboxysome and truncated CsoSCA constructs were used to infer interactions between CsoSCA and the shell (Fig. 4*E* and *SI Appendix*, Fig. S12), future work is required to evaluate the validity of this model (Fig. 5*A*).

There might be a functional relevance to the CsoSCA–CsoS1 interaction. It is believed that HCO_3_^−^, the substrate of CsoSCA, transits the central pores of CsoS1 hexamers to reach the α-carboxysome lumen (11, 61). It is therefore feasible that CsoSCA bound to CsoS1 hexamers act as “substrate gate-keepers,” ensuring that HCO_3_^−^ entering the α-carboxysome is rapidly converted into CO_2_. In the proposed binding arrangement (Fig. 5*A*), the active sites of CsoSCA on the protomers facing the shell facet are ~50 Å away from the central pores of CsoS1A (*SI Appendix*, Fig. S16), so HCO_3_^−^ would not have to travel far to encounter a CsoSCA active site once it passes through the CsoS1 pores. Furthermore, the surface area of CsoSCA directly facing the central pore of CsoS1A is positively charged (*SI Appendix*, Fig. S16), which would encourage the passage of negatively charged HCO_3_^−^ through the central CsoS1 pores and into the α-carboxysome. Nevertheless, since a limited amount of CsoSCA is available to bind to the shell facet, diffusion of HCO_3_^−^ to CsoSCA active sites must still occur to some capacity.

Quantitative analysis of the protein stoichiometry in native *H. neapolitanus* α-carboxysomes (32), combined with the assumption that all CsoSCA proteins in the *H. neapolitanus* α-carboxysome form hexamers, leads to an estimation of approximately 20 CsoSCA hexamers per *H. neapolitanus* α-carboxysome. This highlights the remarkable capacity of the CsoSCA hexamers to generate sufficient CO_2_ to support the catalytic activity of more than 400 Rubisco complexes. As α-carboxysomes are loosely icosahedral structures with ~20 shell facets (33, 34, 36, 37), on average, one CsoSCA hexamer is available to bind to each shell facet. The updated model of the α-carboxysome structure illustrates the proposed location and protein interactions of CsoSCA hexamers within the α-carboxysome (Fig. 5*B*). This organization model, wherein each CsoSCA is localized to the internal shell surface and surrounded by Rubisco enzymes, is supported by modeling studies, which theorize that the smaller, 100 to 150 nm size of α-carboxysomes ensures that Rubisco enzymes situated furthest away from shell-associated CsoSCA, i.e., at the innermost region of the carboxysome lumen, would still receive CO_2_ diffusing from peripheral CsoSCA for efficient carbon fixation (62–64).

### Complex Interactions of CsoSCA within the α-carboxysome.

While CsoS2 plays a crucial role in connecting Rubisco to the carboxysome shell, our results indicate that CsoS2 does not contribute to the encapsulation of CsoSCA (Fig. 3 and *SI Appendix*, Figs. S7, S8, and S13). Instead, CsoSCA can be incorporated into the α-carboxysome in the absence of CsoS2, through direct interactions with the shell hexamers, suggesting distinct encapsulation mechanisms of the two types of cargo enzymes in the α-carboxysome. In the β-carboxysome, the long isoform of CcmM (CcmM58) contains an N-terminal γCAL domain that forms interactions with β-carboxysomal CcaA (27). However, CsoS2 in α-carboxysomes lacks sequence similarity to CcmM and does not possess an equivalent γCAL domain, resulting in a lack of interaction between CsoS2 and CsoSCA. Conversely, α-carboxysomes feature a direct interaction between CsoSCA and Rubisco (*SI Appendix*, Fig. S13) (39), which was not observed between CcaA and Rubisco in β-carboxysomes. This discrepancy is reflected in the conservation of CsoSCA-binding residues within Form 1A Rubisco in α-carboxysomes, whereas these residues are not conserved in Form 1B Rubisco in β-carboxysomes (39). These distinctions highlight the distinct evolutionary paths and functional adaptations of α- and β-carboxysomes.

Our results suggest a potential competition between CsoS2 and CsoSCA for incorporation into the minishell (Fig. 3 *B* and *C*). It is possible that potential binding sites of CsoSCA on the inner shell surface may be obscured by CsoS2, which is known to be essential for assembly of the α-carboxysome shell (37, 42, 43, 45). The purpose of this might be to limit the amount of CsoSCA assembled in the α-carboxysome, as CsoSCA is known to be a minor α-carboxysome component (32). If this is the case, then binding of CsoS2 to the shell should take precedence over CsoSCA, which is supported by the observation that minishells coexpressed with CsoSCA and CsoS2 prefer CsoS2 binding (Fig. 3). In structural models of synthetic or native α-carboxysome shells (37, 45), shell assembly by CsoS2 seemingly commences at the vertices and spans out to stabilize the shell facets, which implies that CsoSCA primarily binds at the center of the shell facets where there are less CsoS2 bound (Fig. 5*A*). Indeed, CsoS2 appears to be absent from the central threefold axis of shell facets in α-carboxysomes purified from *Prochlorococcus* MED4 (37). Furthermore, it is likely that more binding sites for CsoSCA exist on the α-carboxysome shell, but which were not readily identified in cryo-EM structures built from averaging processes during data processing (65). Further studies utilizing larger α-carboxysome shells, such as the recently reported midi-shell structures of bigger average diameters and possessing a full set of shell proteins and CsoS2 fragments, may help determine how CsoSCA is incorporated in a more native context (45).

Our results demonstrate that CsoSCA interacts with both the α-carboxysome shell and Rubisco, suggesting that it bridges Rubisco and the α-carboxysome shell (Fig. 5*B*). We find that CsoSCA can be independently encapsulated in both minishells (Figs. 3 and 4) or intact α-carboxysome shells (*SI Appendix*, Fig. S12) that lack Rubisco, backing initial observations of CsoSCA being a shell-associated component of the α-carboxysome (30, 31). Additionally, a recent study showed that CsoSCA forms strong interactions with Rubisco through its flexible N-terminal region (39). We corroborated the CsoSCA–Rubisco interaction by showing that CsoSCA can recruit CbbL subunits to the minishell in the absence of CsoS2 (*SI Appendix*, Fig. S13*B*). Furthermore, when CsoSCA, as a bait protein, is coexpressed with Rubisco and the minishell, Rubisco, and CsoS1A are found to copurify with CsoSCA (*SI Appendix*, Fig. S13*C*). These findings imply that CsoSCA–shell interactions mediate CsoSCA encapsulation, as well as contribute to Rubisco encapsulation, in the α-carboxysome. This provides a different view to the proposed assembly mechanism of Rubisco being a primary interacting hub for CsoSCA assembly in a different study (39), which also reported undetectable binding between CsoSCA and shell proteins in various biochemical assays. Perhaps the key difference is that in our study, coexpression of CsoSCA with the minishell creates a high intracellular concentration of shell proteins accessible to CsoSCA, allowing protein interactions in form in a more biologically relevant context. Conversely, the in vitro experiments performed in the other study used purified shell proteins that were fused to short ubiquitin-like modifier domains to perturb assembly beyond individual shell pentamers/hexamers. As discussed previously, it is very likely that an interface formed from more than one shell hexamer, as depicted in Fig. 5*A*, is needed for a strong interaction between CsoSCA and the shell to occur. Based on the existing data, there are two possible avenues of CsoSCA assembly in the α-carboxysome: Either CsoSCA is recruited to the shell independent of Rubisco, or CsoSCA is recruited together with Rubisco to the shell, by CsoS2. Future work can focus on identifying if there is a preferred route of assembly in α-carboxysomes.

In summary, this study elucidates the homohexameric structure of CsoSCA from *H. neapolitanus,* how its dimers associate and form the hexamer, and its interactions with other components within the α-carboxysome. Our findings provide a framework for understanding the assembly and functional organization of α-carboxysomes, which will benefit the rational design and bioengineering of carboxysome-based nanostructures for biotechnological applications.

## Materials and Methods

### Plasmids.

The oligonucleotides and plasmids used in this study are described and listed in *SI Appendix*, Tables S2 and S3, respectively. The pHnCBS1D plasmid (Addgene, UK) was used as the template for amplification of native gene sequences from the *H. neapolitanus cso* operon. pHnCBS1D was a gift from David Savage (Addgene plasmid #52065; http://n2t.net/addgene:52065; RRID: Addgene_52065) (47). The CloneAmp HiFi PCR Premix (Takara Bio) was used for PCR amplification and DNA cloning performed with Gibson assembly or Golden Gate cloning (New England Biolabs, UK). All assembled plasmids were sequence-verified by Eurofins Genomics and transformed into TOP10 *E. coli* for storage.

### Bioinformatic Analysis.

*Hn*CsoSCA (UniProt ID O85042) and *Cy*CsoSCA (UniProt ID B5ILN4) sequences were aligned with Clustal Omega (66) and visualized with ESPript 3.0 (67).

Protein sequences of candidate *csoSCA* genes (pfam08936) encoded in finished and permanent draft bacterial genomes within α-carboxysome loci, defined as *cbbL* (pfam00016), *cbbS* (pfam00101), *csoS2* (pfam12288), *csoS1* (pfam00936), and *csoS4* (pfam03319) within 5,000 bp of each other, were collected from the Integrated Microbial Genomes and Microbiomes database on 8 July 2024 using the Cassette Search function (68). This dataset was manually inspected to confirm the presence of α-carboxysome genes and carboxysome loci located at edges of scaffolds were removed. Additionally, *csoSX* from members of *Thiomicrospira* identified as weakly homologous to *csoSCA* but lacking residues for catalytic activity (25, 41) were also removed. Following this, CsoSCA sequences that were >98% identical were removed with Jalview to reduce redundancy, resulting in a final dataset of 333 sequences (Dataset S1). Sequences were aligned with Clustal Omega and visualized with WebLogo (69).

For phylogenetic analysis, the 333 CsoSCA sequences were analyzed based on the absence or presence of the conserved His. Generally, members of the same species are conserved in the absence/presence of the conserved His, therefore one representative sequence of each species was selected. For species with members differing in conservation of the His, one representative sequence with the conserved His and one without were chosen from each species. Additional representative sequences were also selected for *Halothiobacillus* and cyanobacterial CsoSCA. The final dataset was reduced down to 74 sequences. 15 CsoSCA2 sequences (as defined in ref. 41) were selected as the outgroup and added to the list of 74 CsoSCA sequences. Sequence alignment was performed on Clustal Omega and columns with >90% gaps subsequently removed using TrimAI on the Phylemon 2.0 webserver (70). The resulting alignment file was submitted to W-IQ-TREE for inference of a phylogenetic tree by maximum likelihood (71). The best-fit model determined by ModelFinder was LG+F+I+G4. Branch supports were estimated with 1,000 ultrafast bootstrap replicates. The phylogenetic tree was visualized and annotated on iTOL (72).

### Protein Expression and Purification.

Expression and purification of TwinStrep-GB1-CsoSCA/CsoSCA-N/CsoSCA^∆C^/CsoSCA-C: *E. coli* BL21(DE3) harboring the pETM11::Twin-Strep-GB1-CsoSCA/CsoSCA-N/CsoSCA^∆C^/CsoSCA-C construct was grown in 2× Yeast Extract Tryptone medium supplemented with 50 μg mL^−1^ kanamycin, at 37 °C to an OD_600_ of 0.8 to 1.0. To induce protein expression, 0.5 mM isopropyl ß-D-1-thiogalactopyranoside (Melford) was added and cells cultured at 25 °C for 16 h. Cells were harvested at 5,000× g for 10 min and resuspended in Buffer W (100 mM Tris/HCl, pH 8, 150 mM NaCl, 1 mM ethylenediaminetetraacetic acid [EDTA]) supplemented with 10% (v/v) CelLytic™ B cell Lysis Reagent (Sigma-Aldrich) and 0.1% (v/v) Protease Inhibitor Cocktail (Sigma-Aldrich). Cell lysis was performed on a MSE 8-75 MK2 sonicator at six cycles of 30 s ON/OFF, or on the Q125 Sonicator (Qsonica) for 14 min total of 30 s ON/OFF. The lysate was clarified at 27,000× g, 30 min, 4 °C, and supernatant filtered with a 0.45 μm syringe filter. The flow-through was loaded onto a prepacked Strep-TactinXT 4Flow gravity flow column (5 mL bed volume; Thermo Fisher Scientific) pre-equilibrated with 10 mL Buffer W. Column flow-through was collected and reapplied onto the column. The column was then washed with five column volumes (CV) Buffer W followed by addition of 0.6 CV, 1.6 CV, and 0.8 CV of 1× Buffer BXT (diluted from 10× Buffer BXT; Thermo Fisher Scientific), in this order, to elute protein. The 1.6 CV and 0.8 CV fractions were found to contain highly pure and concentrated protein.

For cryo-EM, eluted TwinStrep-GB1-CsoSCA was further purified with a HiLoad 16/600 Superdex 200 pg column (Cytiva) equilibrated in Buffer W. Fractions containing purified protein were pooled and concentrated with 30K MWCO spin concentrators. For SEC-MALS, eluted TwinStrep-GB1-CsoSCA was buffer exchanged into Buffer W with PD-10 desalting columns (Cytiva). For DLS, 15 µg of eluted TwinStrep-GB1-CsoSCA was mixed with 1 µL of tobacco etch virus (TEV) protease (New England Biolabs) in 5 µL of 10× Protease Reaction Buffer and incubated for 3 h on ice.

For SEC-MALS of TwinStrep-GB1-CsoSCA-N, TwinStrep-GB1-CsoSCA^∆C^, and TwinStrep-GB1-CsoSCA-C (*SI Appendix*, Fig. S4), eluted protein from Strep-tag affinity chromatography was concentrated down to <5 mL with 3K MWCO spin concentrators if necessary. Concentrated samples were applied onto a HiLoad 16/600 Superdex 200 pg column (Cytiva) equilibrated in Buffer W + 10% glycerol. Fractions containing purified protein were pooled and concentrated with 3K MWCO spin concentrators.

Expression and purification of mini-α-carboxysome shells (mini-α-shells): Expression and isolation of mini-α-shells were adapted from a protocol that was previously described (42). TOP10 *E. coli* (co-)transformed with the relevant constructs were cultured in 2× Yeast Extract Tryptone medium supplemented with 100 μg mL^−1^ ampicillin, and 10 μg mL^−1^ chloramphenicol where necessary, at 37 °C to an OD at 600 nm (OD_600_) of 0.8 to 1.0. Protein expression was induced with 1 mM arabinose (Melford) at 22 °C for 16 h. Cells were pelleted at 5,000× g for 10 min and resuspended in TEMB buffer (10 mM Tris/HCl pH 8, 1 mM EDTA, 10 mM MgCl_2_, 20 mM NaHCO_3_) supplemented with 10% (v/v) CelLytic™ B cell Lysis Reagent (Sigma-Aldrich) and 0.1% (v/v) Protease Inhibitor Cocktail (Sigma-Aldrich). Cells were lysed by sonication using a MSE 8-75 MK2 sonicator, at six cycles of 30 s ON/OFF and clarified at 27,000× g, 30 min, 4 °C. Supernatant was loaded on top of 5 mL 30% (w/v) sucrose and minishells pelleted at 250,000× g, 16 h, 4 °C. Pellets were carefully resuspended with a soft brush and further centrifuged at 20,000× g, 2 min, 4 °C. Supernatant was applied on top of an 8 mL step sucrose gradient consisting of 20%, 30%, 40%, and 50% (w/v) sucrose layers and centrifuged at 70,000× g, 16 h, 4 °C. Each sucrose layer was collected and analyzed with sodium dodecyl sulfate-polyacrylamide gel electrophoresis (SDS-PAGE); the 30% and 40% sucrose fractions were consistently found to be enriched with mini-α-shells. These two fractions were pooled and loaded onto a HiTrap Q FF anion exchange chromatography column (Cytiva Life Sciences) equilibrated with Buffer A (TEMB plus 50 mM NaCl). The column was washed with Buffer A and five CV of 20% Buffer B (TEMB plus 1 M NaCl), followed by a linear gradient of 20 to 40% Buffer B over 10 CV to elute the mini-α-shells. CsoSCA was found to elute at 20% Buffer B, whereas minishell elution was typically observed at 30 to 35% Buffer B. Fractions containing purified mini-α-shells were identified by SDS-PAGE and negative-staining TEM, followed by sample concentration and buffer exchange into TEMB with 0.5 mL 100K MWCO Pierce™ Protein Concentrators PES (Thermo Fisher Scientific). Purified protein was stored at 4 °C for further analysis. To optimize the minishell purification for isolation of more *T* = 4 shells, 30% and 40% sucrose fractions were separately loaded onto the HiTrap Q FF anion exchange chromatography column.

Expression and purification of α-carboxysomes (α-CB) and α-shells: Expression and isolation of α-CB and α-shells were adapted from a protocol that was previously described (31). TOP10 *E. coli* cells transformed with the relevant constructs were cultured in 2× Yeast Extract Tryptone medium, supplemented with 100 μg mL^−1^ ampicillin, at 37 °C to an OD_600_ of 0.8 to 1.0. Protein expression was induced with 1 mM arabinose (Melford) at 25 °C for 16 h. Cells were pelleted at 5,000× g for 10 min and resuspended in TEMB buffer supplemented with 10% (v/v) CelLytic™ B cell Lysis Reagent (Sigma-Aldrich) and 0.1% (v/v) Protease Inhibitor Cocktail (Sigma-Aldrich). Cells were lysed by sonication using a MSE 8-75 MK2 sonicator, at 12 cycles of 30 s ON/OFF (performed in two six cycle batches with an incubation on ice in between) and clarified at 12,000× g, 10 min, 4 °C. Supernatant was centrifuged at 47,000× g, 30 min, 4 °C to pellet carboxysomes. Pellets were carefully resuspended with a soft brush and applied on top of a 10 mL step sucrose gradient consisting of 10%, 20%, 30%, 40%, and 50% (w/v) sucrose layers, followed by centrifugation at 105,000× g, 30 min, 4 °C. Each sucrose layer was collected and analyzed with SDS-PAGE and negative-staining TEM. The 30% sucrose fractions from both the α-CB and α-shell purifications had the highest sample purity and good yield and were selected for further analysis.

### In Vivo Pull-Down Assay.

Bait and prey proteins were (co-)expressed and harvested as described in the previous section. Cell pellets were resuspended in Buffer W and cell lysis performed on a MSE 8-75 MK2 sonicator, at six cycles of 30 s ON/OFF. The lysate was clarified at 27,000× g, 30 min, 4 °C. Empty gravity flow columns (Thermo Fisher Scientific) were handpacked with StrepTactinXT 4Flow (1 mL bed volume; Thermo Fisher Scientific) for StrepII-CsoSCA-His_6_ coexpressed with minishells (*SI Appendix*, Fig. S8), or StrepTactin XT 4Flow high capacity (0.5 mL bed volume; Thermo Fisher Scientific) for StrepII-CsoSCA coexpressed with minishells and Rubisco (*SI Appendix*, Fig. S13). Clarified protein lysates were filtered through a 0.45 μm filter before applying onto their respective columns, which were pre-equilibrated with 2 CV Buffer W. The columns were washed with at least 10 CV of Buffer W followed by addition of 0.6 CV, 1.6 CV, and 0.8 CV of 1× Buffer BXT (diluted from 10× Buffer BXT; Thermo Fisher Scientific), in this order, to elute protein. The 1.6 CV fraction was used for the detection of bait and prey protein by Western immunoblotting.

### Immunoblot Analysis.

Protein samples were prepared and resolved by SDS-PAGE as previously described (42). Proteins were probed with anti-CsoS1A/B/C (Agrisera, Cat No. AS14 2760, 1:5,000 dilution), anti-6x His Tag (Invitrogen, Cat No. MA1-135, 1:5,000 dilution), anti-CsoS2-C (synthesized by PhytoAB with the peptides APRSDQMDRVSGEGK and FANRNVPKPEKPGSK, 1:10,000 dilution), anti-RbcL (Agrisera, Cat No. AS03 037, 1:10,000 dilution), anti-CsoS2-N (synthesized by GenScript, NJ, USA with the RGTRAVPPKPQSQG peptide, 1:10,000 dilution), anti-CsoSCA (prepared by Genscript, NJ, USA, using the RHGGRYPPNDIGHA peptide, 1:1,000 dilution), horseradish peroxidase-conjugated goat anti-rabbit immunoglobulin G secondary antibody (Agrisera, Cat No. AS09 602, 1:10,000 dilution) and horseradish peroxidase-conjugated goat anti-mouse immunoglobulin G secondary antibody (Agrisera, Cat No. AS11 1772, 1:5,000 dilution). The Bio-Rad chemiluminescence kit (Bio-Rad, UK) was used for detection on a ImageQuant LAS 4000 (GE Healthcare, USA). Peak intensities of the protein bands of interest were measured on Fiji software (73) for relative protein quantification.

### SEC-MALS.

TwinStrep-GB1-CsoSCA (5.6 mg mL^−1^), TwinStrep-GB1-CsoSCA-N (0.9 mg mL^−1^), TwinStrep-GB1-CsoSCA^ΔC^ (1 mg mL^−1^), and TwinStrep-GB1-CsoSCA-C (1.9 mg mL^−1^) were respectively injected onto a Superdex 200 Increase 10/300 GL (Cytiva) at a flow rate of 0.70 mL min^−1^ in Buffer W at room temperature. The column was connected to an ÄKTA pure chromatography system (Cytiva) coupled with a DAWN 8+ multiangle light scattering detector (Wyatt Technology) and a Optilab T-rEX refractive index detector (Wyatt Technology). Bovine serum albumin (ThermoFisher) was used as the calibration standard. Molecular masses were calculated with ASTRA 6.1 software (Wyatt Technology). The graphs shown in *SI Appendix*, Figs. S1*D* and S4 were plotted on Microsoft Excel.

### Dynamic Light Scattering.

TwinStrep-GB1-CsoSCA or CsoSCA (after TEV cleavage) were respectively loaded into nanoDSF capillaries carefully. Size analysis measurements were performed on a Prometheus Panta nanoDSF instrument (NanoTemper Technologies, Germany) at 20 °C. Data analysis was performed on Panta Control analysis software v1.6.3.

### Negative-Staining TEM.

Carbon films on 300 Mesh Grids Copper (Agar Scientific, UK) were glow discharged. 5 µL of purified protein sample (A_280nm_ ~0.5 to 1) were mounted on the grid for 40 s, then stained and washed with 60 µL of 2% uranyl acetate (Sigma-Aldrich). Excess stain was wicked away with filter paper and grids left to air dry for at least 1 min. Grids were imaged with a FEO Tecnai G2 Spirit Bio TWIN microscope equipped with a Gatan Rio 16 camera. Images were visualized and analyzed with Fiji software (73). *SI Appendix*, Fig. S9 was plotted on Origin (Origin, Version 2024b, OriginLab Corporation, Northampton, MA, USA).

### Cryo-EM Sample Preparation.

3 µL of purified samples were applied to Quantifoil Cu200 R1.2/1.3 grids that were glow discharged for 60 to 120 s at 20 mA on a PELCO easiGlow. Grids were blotted at 4 °C with 100% humidity and plunge frozen into liquid ethane using a Vitrobot Mark IV (Thermo Scientific). For data acquisition, grids were screened for optimal ice thickness and particle distribution on a 200 kV Glacios cryotransmission EM (cryo-TEM) equipped with a Falcon IV direct electron detector at the University of York.

### Cryo-EM Data Acquisition.

Data collection of *Hn*CsoSCA and the minishell/CsoSCA-C dataset was performed on a 200 kV Glacios cryo-TEM equipped with a Falcon IV direct electron detector. A total of 7,997 and 2,198 movie stacks *Hn*CsoSCA and minishell/CsoSCA-C were respectively collected with EPU at a total dose of 50 e^−^Å^−2^ in a defocus range of −0.6 to −2.0 μm. The nominal magnification was ×240,000 corresponding to a pixel size of 0.574 Å. The dataset to generate the high-resolution minishell/CsoSCA^∆C^ map was collected on a 300 kV Titan Krios microscope equipped with a BioQuantum K3 direct electron detector at the UK’s National electron Bio-Imaging Centre (eBIC). A total of 9,998 movie stacks were collected with EPU at a total dose of 50 e^−^Å^−2^ in a defocus range of −0.6 to −2.0 μm. The nominal magnification was ×105,000 corresponding to a pixel size of 0.825 Å.

### Cryo-EM Data Processing.

Data processing was performed using cryoSPARC 4.3 (74). *SI Appendix*, Fig. S2 (*Hn*CsoSCA), *SI Appendix*, Fig. S10 (minishell/CsoSCA^∆C^), and *SI Appendix*, Fig. S11 (minishell/CsoSCA-C) show the data processing pipelines. Data collection and processing statistics are summarized in *SI Appendix*, Table S1. Collected movie stacks were patch motion-corrected and patch contrast transfer function (CTF) parameters were estimated.

For *Hn*CsoSCA, particles were picked with blob picker (70 to 150 Å) and subjected to successive rounds of 2D classification for generation of an ab initio model after fewer than 5% of particles were rejected per round of 2D classification. The ab initio model was homogeneously refined with C1 followed by D3 symmetry to be used as a template for autopicking. The new set of particles was used to enhance the resolution of the initial volume and global and local CTF refinements performed. The volume was then put through an ab initio job set to two classes to remove junk particles. The good class of particles was CTF refined and a round of reference-based motion correction was performed on the particle set before a final round of homogeneous refinement.

Using preliminary datasets of minishell/CsoSCA^∆C^ and minishell/CsoSCA-C, blob picking identified two distinct minishell symmetries, *T* = 3 and *T* = 4, which were independently processed. No additional internal density or shell-associated peptide were observed in the *T* = 3 shells for either dataset after the final refinement step. Particle subtraction of the shell density and nonuniform refinement failed to tease out any internal density for the *T* = 3 shells. Ab initio and homogeneous refinements of both datasets with a loose spherical mask and lower-order symmetries imposed, including C1, C2, C3, C5, and D2, revealed the presence of internal density that was highly unstructured. Therefore, processing of the *T* = 3 shells was not taken further. In contrast, *T* = 4 shells in both datasets with I symmetry applied shows the appearance of internal density at low contour levels which looked comparatively more structured. More data were collected for the minishell/CsoSCA^∆C^ and minishell/CsoSCA-C samples, with data processing focused on the *T* = 4 shells to attempt to gain a better reconstruction of the internal density.

For the minishell/CsoSCA^∆C^ dataset, *T* = 4 particles picked with blob picker (200 to 250 Å) were subjected to consecutive rounds of 2D classification. The final 2D classes were used to generate an ab initio template with no imposed symmetry, followed by homogeneous and CTF refinements with icosahedral symmetry imposed. More particles were picked to improve the resolution of the map, before subjecting the data to CTF, Ewald Sphere and reference-based motion corrections. A spherical mask was used to ensure any internal density was not masked off by automasking. The final 1.82 Å map was used to build an atomic model of the minishell using the previously published minishell model as a starting model (PDB: 8B11). To tease out the internal density, this map was symmetry relaxed to generate a 2.36 Å map. A similar processing pipeline was used for the minishell/CsoSCA-C dataset. Blob picker (220 to 300 Å) was used to pick minishells, and *T* = 4 shells selected in successive rounds of 2D classification. More particles were picked using template picker and duplicates removed. This set of particles were used for ab initio reconstruction with enforced icosahedral symmetry followed by homogeneous and CTF refinements, producing a final map at 3.05 Å.

### Model Building and Refinement.

#### HnCsoSCA.

Model refinement statistics are summarized in *SI Appendix*, Table S1. A starting model of a *Hn*CsoSCA protomer was built with ModelAngelo (75) using the final cryo-EM density map, a mask covering a single asymmetric unit, and the *Hn*CsoSCA amino acid sequence. The initial model was then manually examined and adjusted in Coot (76) to fit the density map. Subsequent refinements of the model were performed iteratively with real-space refinement in Phenix (77) and adjusted in Coot. To build the hexameric model, the Phenix Map Symmetry tool was run to determine map symmetry and Phenix Apply NCS operators applied to fit the protomer into all six monomers in the hexamer. All structural figures were prepared with UCSF ChimeraX (78). Interactions at the dimer and hexamer interface were analyzed with PDBePISA (https://www.ebi.ac.uk/pdbe/pisa/) and LigPlot v2.2.8 (79).

#### Minishells.

For both minishell/CsoSCA^∆C^ and minishell/CsoSCA-C, the starting model of the *T* = 4 asymmetric unit was derived from PDB 8B11. Density corresponding to the atomic model was cut out with the Map Box tool in Phenix for further iterations of real-space refinement in Phenix and manual adjustments in Coot to build the final model.

## Supplementary Material

Appendix 01 (PDF)

Dataset S01 (XLSX)

## Data Availability

Cryo-EM density maps have been deposited in the Electron Microscopy Data Bank (EMDB) under accession codes EMD-51067 (*Hn*CsoSCA) (80), EMD-51633 (minishell/CsoSCA^∆C^) (81), and EMD-51641 (minishell/CsoSCA-C) (82). The corresponding atomic models have been deposited in the Protein Data Bank (PDB) under the accession codes 9G4T (*Hn*CsoSCA) (83), 9GVC (minishell/CsoSCA^∆C^) (84), and 9GW1 (minishell/CsoSCA-C) (85).
