# Supplementary material for: Structure and encapsulation of carbonic anhydrase within the α-carboxysome
Source: Proc Natl Acad Sci U S A. 2025 Nov 12;122(46):e2523723122. doi: 10.1073/pnas.2523723122 (PMC12646314; doi:10.1073/pnas.2523723122)
Supplement: Supplementary file 1 — Appendix 01 (PDF) [file pnas.2523723122.sapp.pdf]

PNAS

Supporting Information for

Structure and encapsulation of carbonic anhydrase within the α-carboxysome

Pei Cing Ng[1], Oluwatobi Adegbite[1], Tianpei Li[1], Arnaud Basle[2], Jon Marles-Wright[2]*, Lu-Ning Liu[1,3]*

Corresponding authors: Lu-Ning Liu; Jon Marles-Wright
Email: luning.liu@liverpool.ac.uk (L.-N.L.); Jon.Marles-Wright1@newcastle.ac.uk (J.M.W.)

This PDF file includes:

Figures S1-16
Tables S1-3
SI References

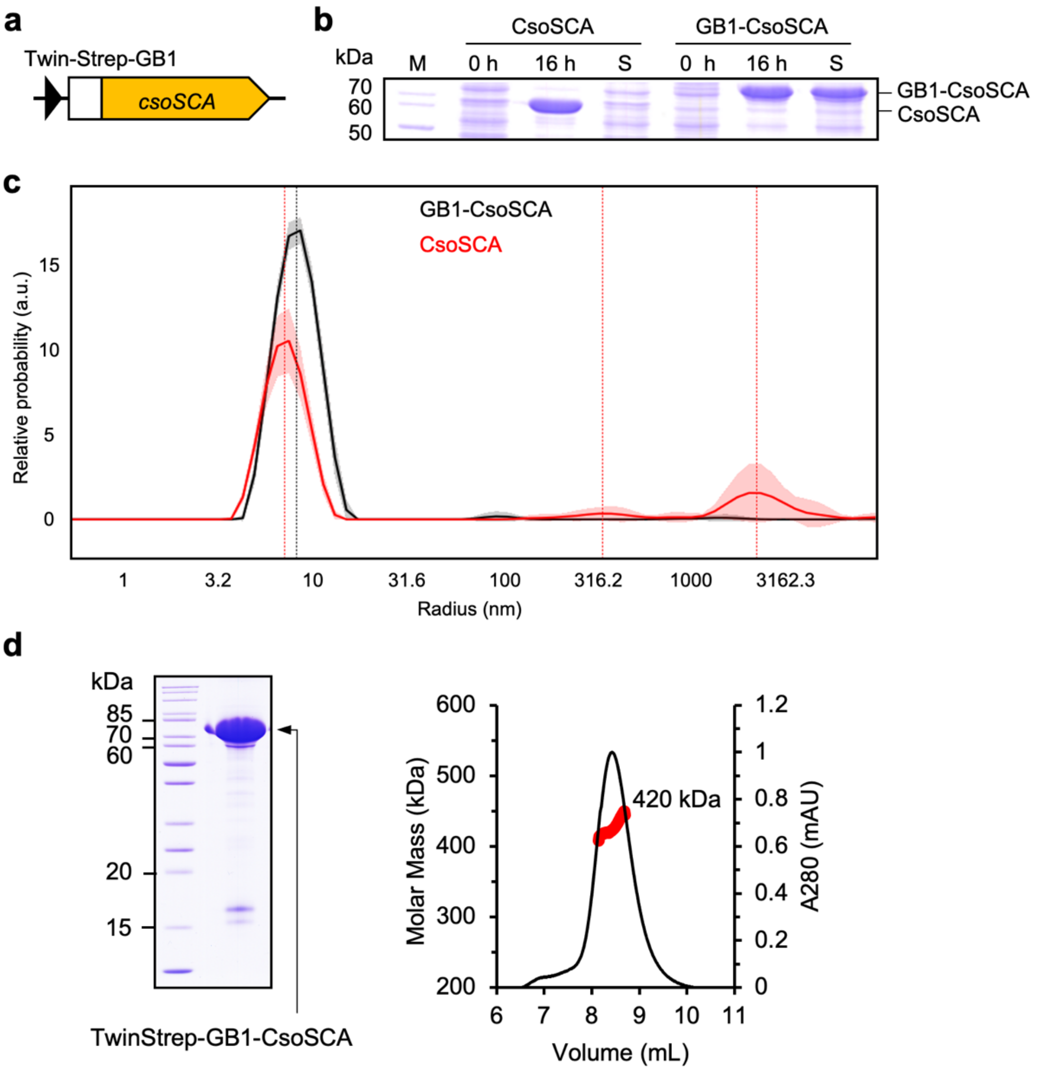

Supplementary Figure 1. Expression and purification of recombinant *Hn*CsoSCA. a, Schematic of the Twin-Strep-GB1-CsoSCA construct used for purification of *Hn*CsoSCA. **b,** SDS-PAGE of supernatant (S) and whole cell lysate samples collected before (0 h) and after (16 h) IPTG induction of Twin-Strep-CsoSCA (CsoSCA) and Twin-Strep-GB1-CsoSCA (GB1-CsoSCA). **c,** Dynamic light scattering of CsoSCA (red) and GB1-CsoSCA (black) at 20˚C. Aggregation is observed for CsoSCA. a.u., arbitrary units. **d,** Left: SDS-PAGE of Twin-Strep-GB1-CsoSCA purified by Strep-tag affinity chromatography. Right: The sample elutes as a single 420 kDa species on SEC-MALS. The measured molecular weight is shown as a horizontal red line. The expected molecular weight of the Twin-Strep-GB1-CsoSCA hexamer is 408.6 kDa.

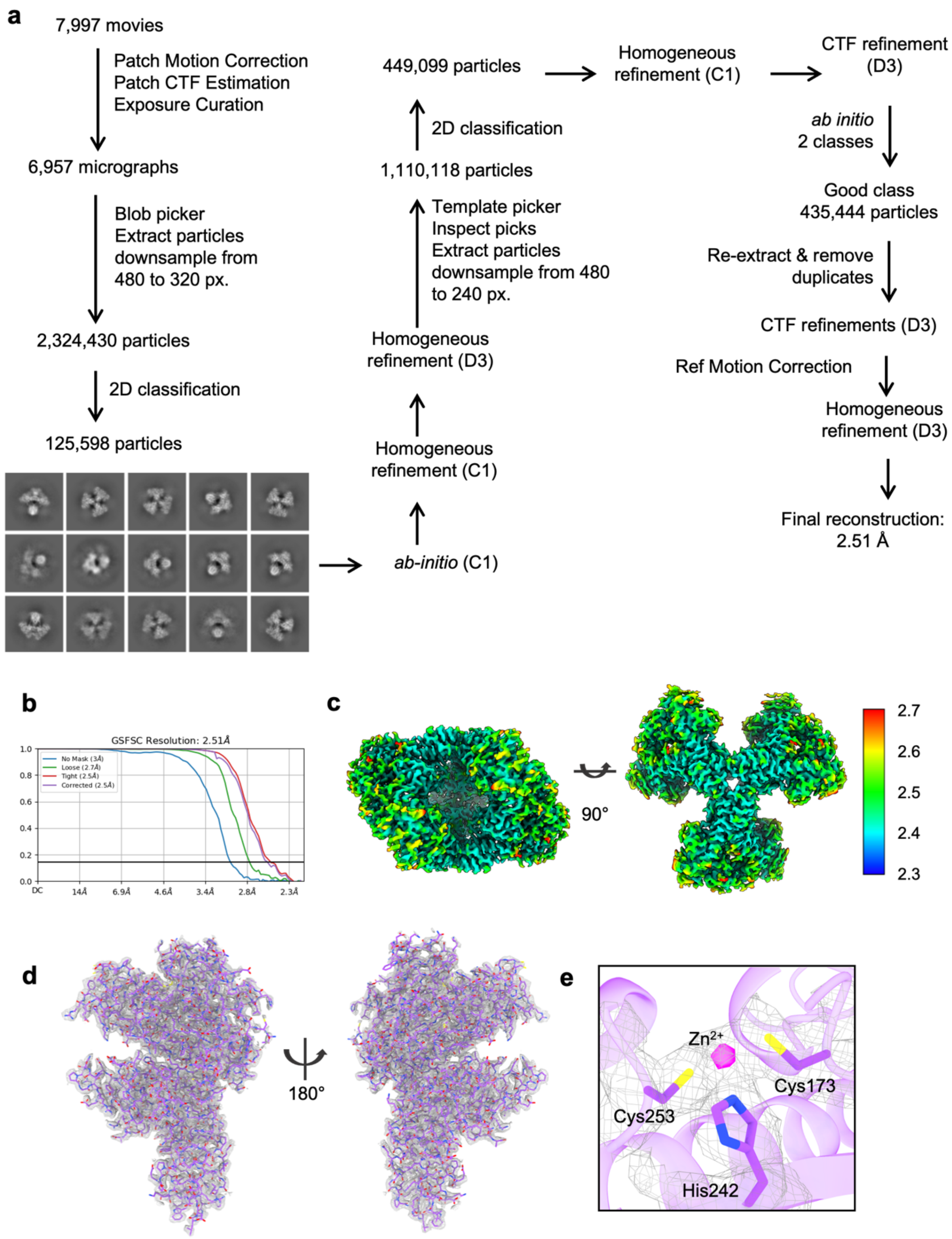

Supplementary Figure 2. Data processing of the *Hn*CsoSCA hexamer on cryoSPARC. a, Cryo-EM data processing workflow for the *Hn*CsoSCA dataset. Representative 2D class averages are shown in the schematic. **b,** FSC curve of *Hn*CsoSCA. **c,** Local resolution map of *Hn*CsoSCA showing the high resolution of the hexamer interface. **d,** Cryo-EM density map fitted with a single protomer of the *Hn*CsoSCA model. **e,** The catalytic zinc (magenta sphere) is coordinated by Cys173, His242 and Cys253 at the catalytic site of *Hn*CsoSCA.

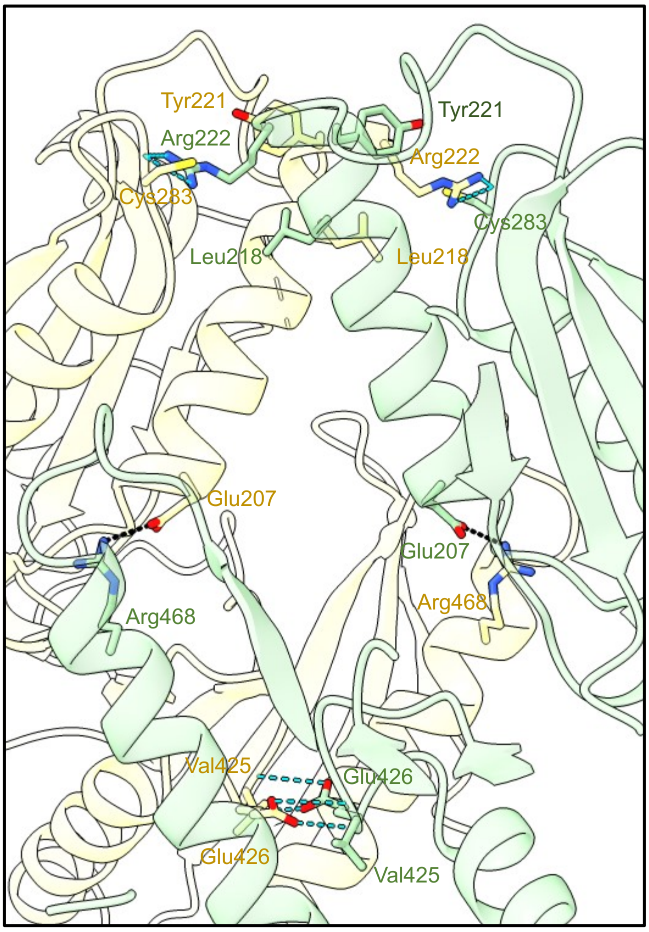

Supplementary Figure 3. Polar interactions at the dimer interface of *Hn*CsoSCA. Two protomers of the same dimer in *Hn*CsoSCA are represented as yellow and green cartoons respectively. Residues identified to mediate polar interactions are shown as sticks and labelled. Blue and black dashed lines depict hydrogen bonding and salt bridges, respectively.

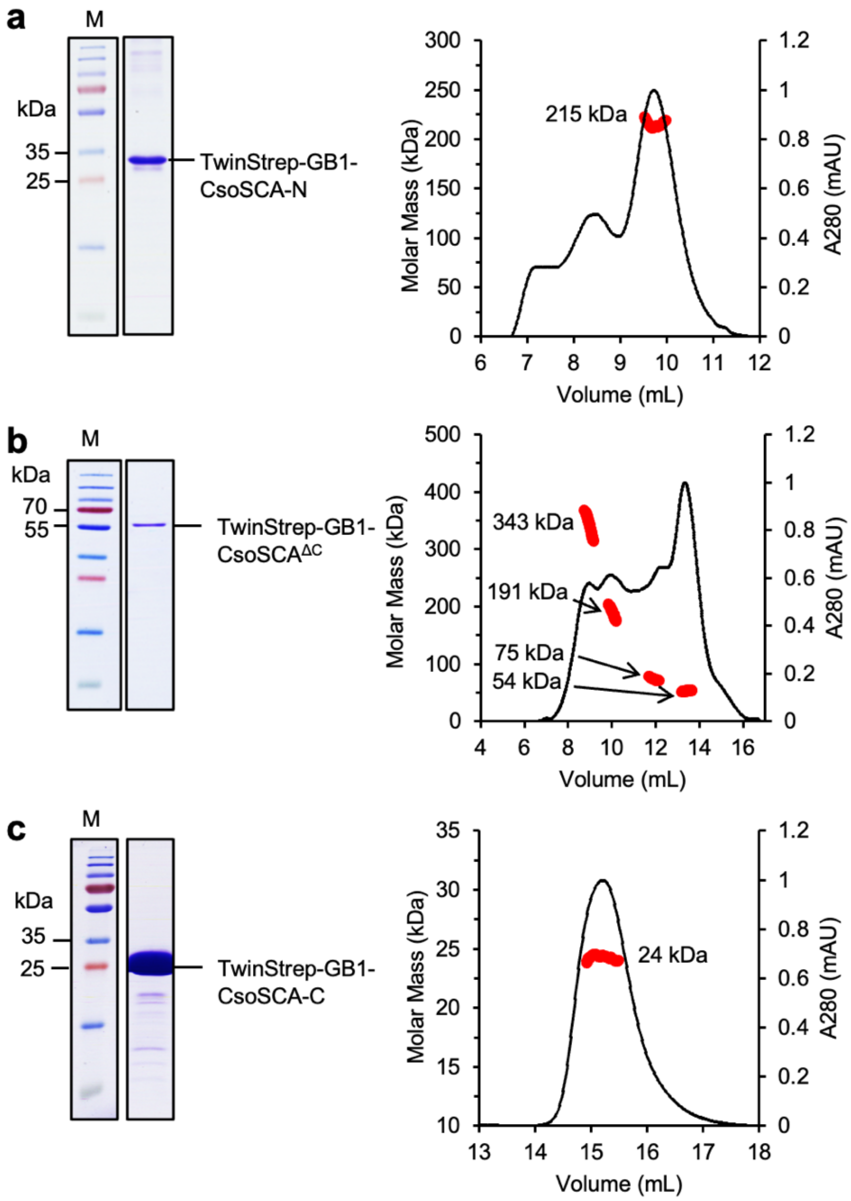

Supplementary Figure 4. SEC-MALS of truncated CsoSCA variants. SDS-PAGE (left) and SEC-MALS (right) profiles of purified Twin-Strep-GB1-CsoSCA-N (**a**), TwinStrep-GB1-CsoSCA$^{\Delta C}$ (**b**) and TwinStrep-GB1-CsoSCA-C (**c**). The measured molecular masses of the CsoSCA truncations on SEC-MALS are shown as horizontal red lines. The expected monomeric molecular masses of Twin-Strep-GB1-CsoSCA-N, TwinStrep-GB1-CsoSCA$^{\Delta C}$ and TwinStrep-GB1-CsoSCA-C are 27.5 kDa, 54.8 kDa and 24.1 kDa, respectively.

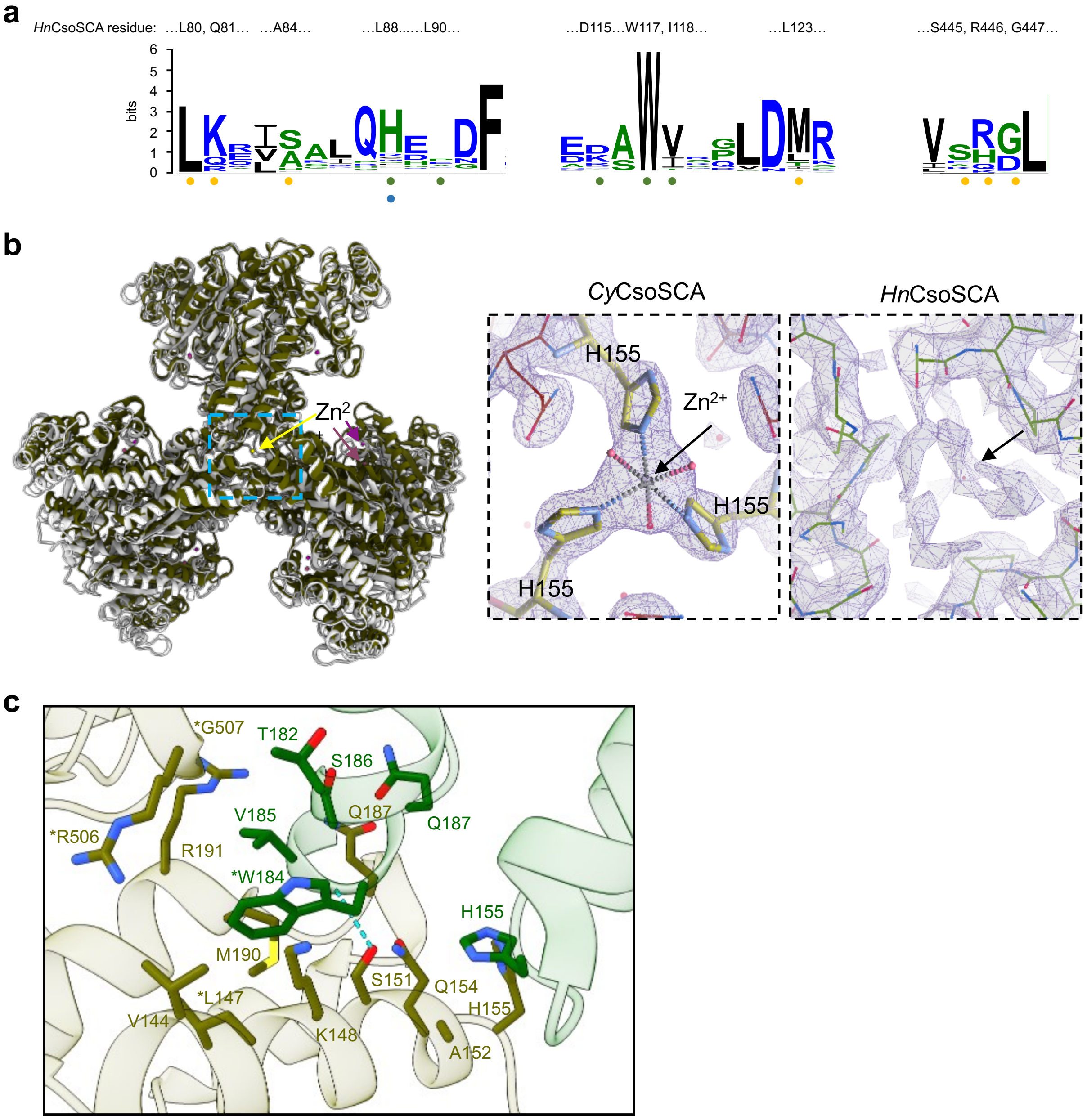

Supplementary Figure 5. Forces driving hexamerization of CsoSCA. a, WebLogo conservation plots of residues mediating *Hn*CsoSCA and *Cy*CsoSCA hexamerization in 333 CsoSCA sequences associated with α-carboxysomes. Residues involved in *Hn*CsoSCA oligomerization shown in Fig. 2a are annotated with yellow and green circles. His155 of *Cy*CsoSCA is marked by a blue circle. Residue numbering above the alignment is based on the *Hn*CsoSCA sequence. **b,** Comparison of the hexameric *Cy*CsoSCA (dark green) and *Hn*CsoSCA (white) structure. *Cy*CsoSCA was superimposed to *Hn*CsoSCA using the MatchMaker tool in ChimeraX. The structural zinc of *Cy*CsoSCA (yellow), as well as the catalytic zinc of *Hn*CsoSCA (light pink) and *Cy*CsoSCA (dark purple) are shown. Left inset shows a zoomed in view of the structural zinc (gray sphere) and its octahedral coordination sphere of *Cy*CsoSCA, comprising of His155 (yellow sticks) from three distinct protomers and three H_2O molecules (red spheres). The electron density map of *Cy*CsoSCA converted from validated $2F_o$ - F_c coefficients is shown at 2.10 σ. Right inset shows the corresponding site in *Hn*CsoSCA, where there is a lack of density corresponding to a coordination sphere. The *Hn*CsoSCA density map is shown at 4.00 σ. Images shown in the insets were generated in Coot. **c,** Residues involved in hydrophobic and hydrogen bonding interactions at the *Cy*CsoSCA hexameric interface. Different colors denote the two protomers of the interface. The dashed cyan line represents the hydrogen bond between the hydroxy group of Ser151 and main chain carbonyl group of Trp184. Residues marked with an asterisk were found to be highly conserved in α-carboxysomal CsoSCA as shown in **a.**

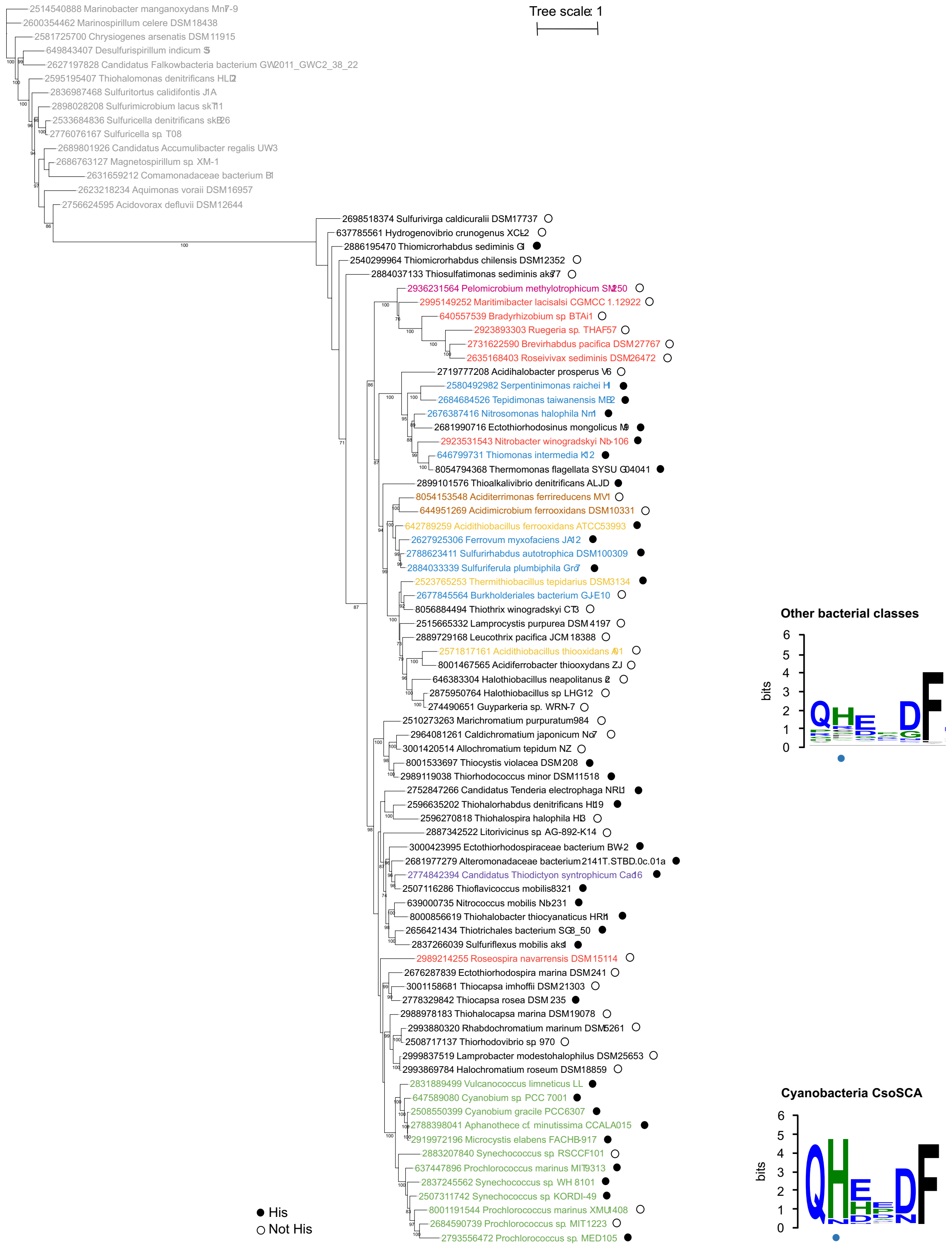

Supplementary Figure 6. Conservation of His155 *Cy*CsoSCA in α-carboxysomal CsoSCA is dependent on lineage. Maximum likelihood analysis of CsoSCA from α-carboxysome loci. Leaves are labelled with the full organism name and the IMG gene ID of its *csoSCA*. Black circle denotes conservation of His; white circle denotes no conservation of His. Gray sequences: CsoSCA2; black sequences: γ-proteobacteria; pink sequences: Hydrogenophilialia; red sequences: α-proteobacteria; blue sequences: β-proteobacteria; brown sequences: Actinobacteria; yellow sequences: Acidithiobacilla; purple sequences: purple bacteria; green sequences: cyanobacteria. Scale bar refers to number of substitutions per site. WebLogo plots of the conserved His (denoted by blue circle) in 146 cyanobacterial CsoSCA and 173 CsoSCA sequences from other bacterial classes. His conservation is found in ~91% of cyanobacterial CsoSCA and ~49% of CsoSCA from other bacterial classes.

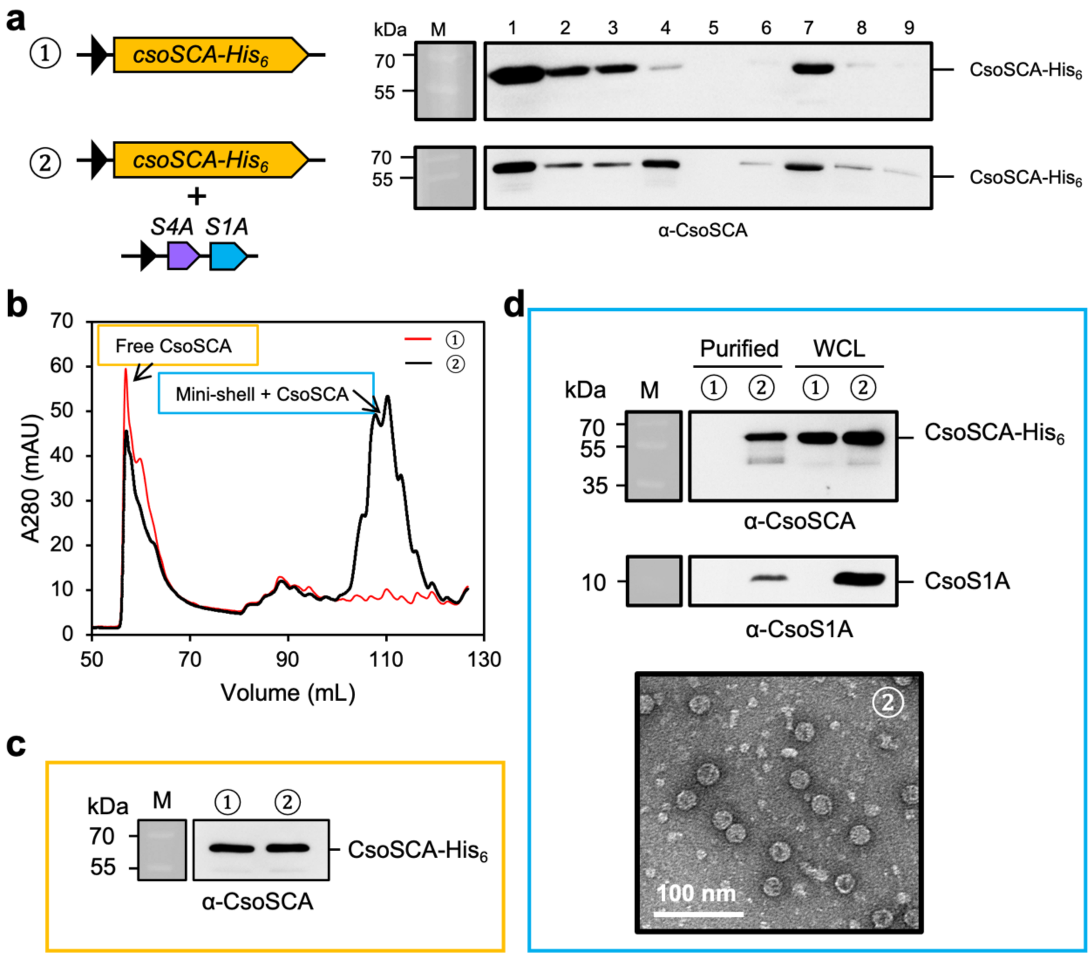

Supplementary Figure 7. Isolation of CsoSCA compared to CsoSCA co-expressed with the mini-shell. **a,** Left: Schematic representation of the experimental set-up. CsoSCA with a C-terminal 6xHis tag (CsoSCA-His$_6$) was either expressed by itself (1), or co-expressed with the mini-shell (2). Purification of both samples were performed in parallel using the mini-shell isolation protocol. Right: Immunoblotting of samples up to the 20-50% step sucrose gradient. Top blot: sample 1; bottom blot: sample 2. Lanes: 1, whole cell lysate; 2, soluble fraction; 3, supernatant after 30% sucrose ultracentrifugation; 4, resuspended pellet after 30% sucrose ultracentrifugation; 5, sample fraction after 20-50% sucrose ultracentrifugation; 6, 20% sucrose fraction after 20-50% sucrose ultracentrifugation; 7, 30% sucrose fraction after 20-50% sucrose ultracentrifugation; 8, 40% sucrose after 20-50% sucrose ultracentrifugation; 9, 50% sucrose after 20-50% sucrose ultracentrifugation. **b,** Anion exchange chromatography of sample 1 (red line) and sample 2 (black line). The elution fractions of free/unencapsulated CsoSCA-His$_6$ and mini-shells are indicated. **c,** Immunoblotting of the elution fractions of free/unencapsulated CsoSCA-His$_6$ from anion exchange chromatography (b). **d,** Top: elution fraction of mini-shells in sample 2 and the corresponding fraction in sample 1 (purified) from anion exchange chromatography (**b**), as well as the expression profiles of samples 1 and 2 (whole cell lysates, WCL). Bottom: negative-stain transmission EM of purified mini-shells from sample 2.

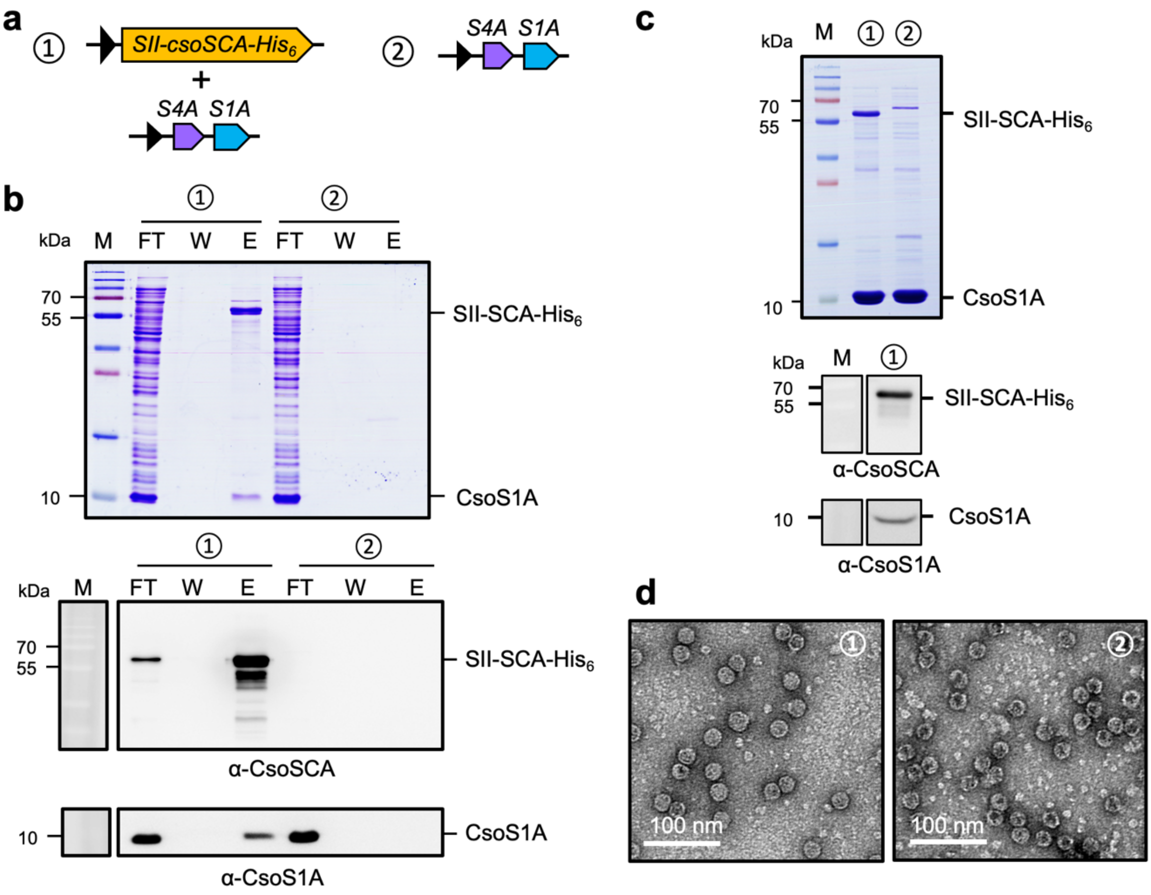

Supplementary Figure 8. Pulldown and isolation of Strep-II tagged CsoSCA co-expressed with mini-shells. a, Schematic representation of the experimental set-up. CsoSCA fused simultaneously to an N-terminal Strep-tag II and a C-terminal 6xHis tag (SII-CsoSCA-His_6) was co-expressed with the mini-shell (sample 1). Expression of the mini-shell without SII-CsoSCA-His_6 (sample 2) was performed as a control. **b,** *In vivo* pulldown assay of sample 1 and sample 2 using SII-CsoSCA-His_6 as the bait protein. The flow-through (FT), final wash step (W) and elution (E) of the pulldown assay were analyzed by SDS-PAGE and immunoblotting. **c,** The flow-through of the *in vivo* pulldown assay was further purified using the mini-shell isolation protocol and isolated mini-shells analyzed by SDS-PAGE and immunoblotting. **d,** Negative-stain transmission EM of purified mini-shells from samples 1 and 2.

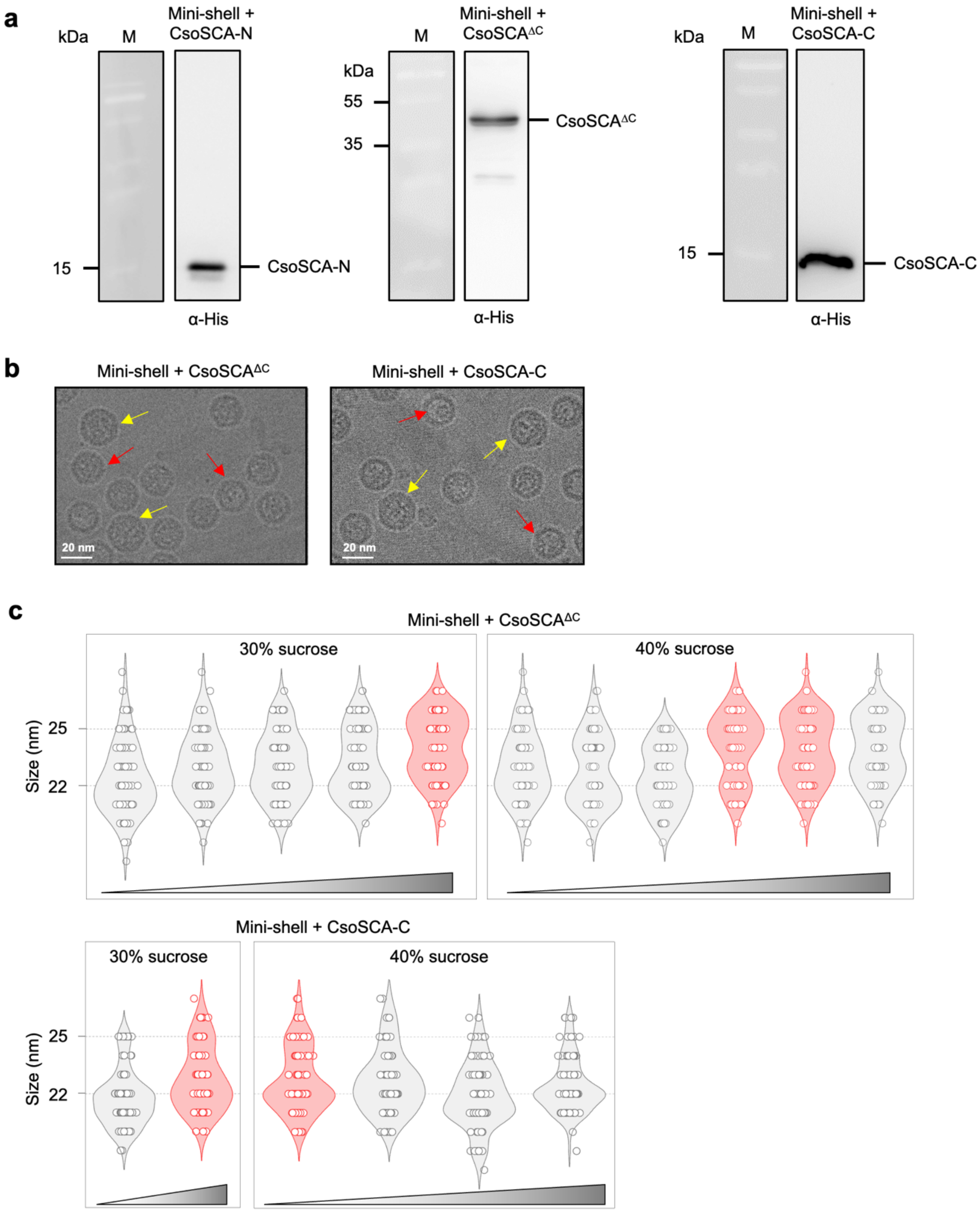

Supplementary Figure 9. Expression and purification of mini-shells containing CsoSCA variants. a, Immunoblotting of the soluble fraction of mini-shell/CsoSCA-N (left), mini-shell/CsoSCA$^{\Delta C}$ (center) and mini-shell/CsoSCA-C (right) samples. CsoSCA variants are tagged with C-terminal His_6 tag. **b,** Representative cryo-EM micrographs of purified mini-shell/CsoSCA$^{\Delta C}$ and mini-shell/CsoSCA-C. Scale bar represents 20 nm. A mixture of *T*=3 (red arrows) and *T*=4 (yellow arrows) mini-shells are observed in both samples. **c,** Violin plots showing the distribution of *T*=3 (22 nm) and *T*=4 (25 nm) mini-shells in elution fractions from anion exchange chromatography. % of Buffer B is represented by the gradient. Samples from elution fractions were visualized with negative-staining TEM and sizes of the shells measured with Fiji. 100 mini-shells (*n* = 100) were measured for each fraction shown; each density curve represents a single fraction. Fractions highlighted in red were pooled and concentrated down for cryo-EM analysis.

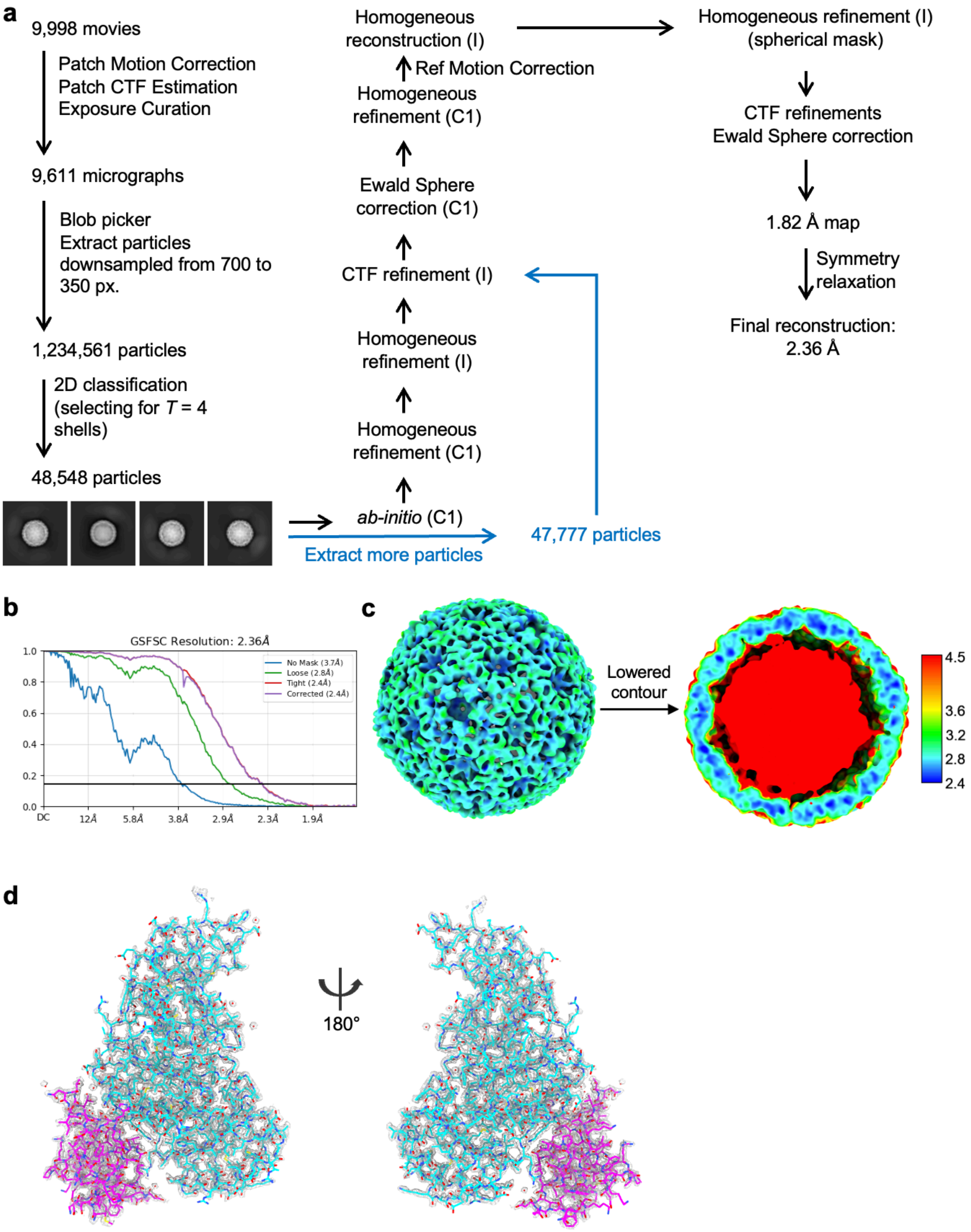

Supplementary Figure 10. Data processing of mini-shell/CsoSCA$^{\Delta C}$ on cryoSPARC. a, Cryo-EM data processing workflow of the mini-shell/CsoSCA$^{\Delta C}$ dataset. Representative 2D classes of the *T*=4 shells are shown in the schematic. The 1.82 Å density map was used to build the atomic model of the shell, whilst the final symmetry relaxed reconstruction at 2.36 Å was used to show the internal density. **b,** FSC curve of mini-shell/CsoSCA$^{\Delta C}$. **c,** Gaussian filtered local resolution map of mini-shell/CsoSCA$^{\Delta C}$ showing the high resolution of the shell and the lower resolution of the internal density. Map gaussian filtered to two standard deviations in ChimeraX; left: density map at high contour (0.045); right: density map at low contour (0.025) and clipped to show internal features. Color representation of the local resolution is shown in the key. **d,** The asymmetric unit of mini-shell/CsoSCA$^{\Delta C}$, consisting of three CsoS1A monomers (cyan) and one CsoS4A monomer (magenta), is fitted to the 1.82 Å density map. Water molecules are shown as red spheres.

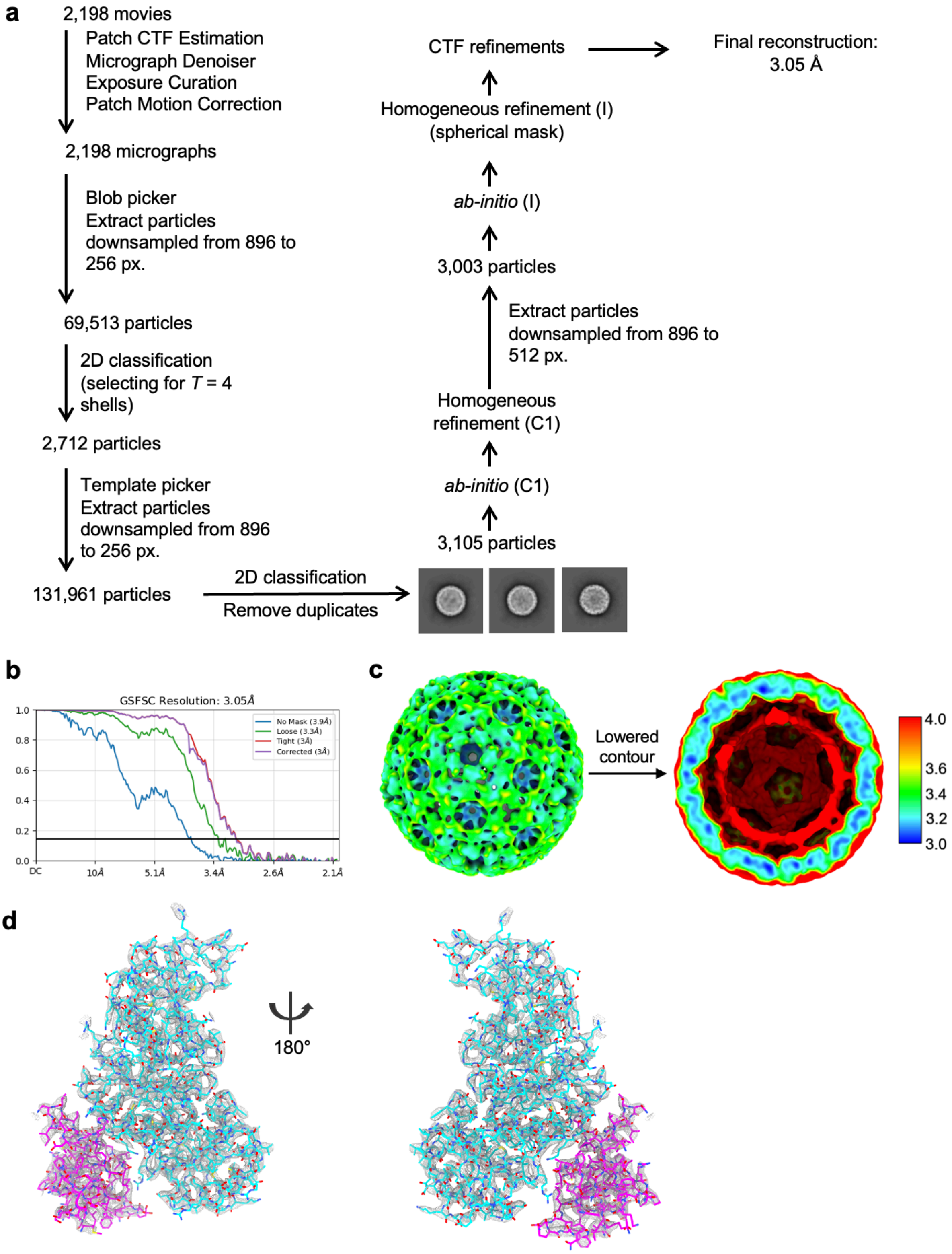

Supplementary Figure 11. Data processing of mini-shell/CsoSCA-C on cryoSPARC. a, Cryo-EM data processing workflow of the mini-shell/CsoSCA-C dataset. Representative 2D classes of the *T*=4 shells are shown in the schematic. **b,** FSC curve of mini-shell/CsoSCA-C. **c,** Gaussian filtered local resolution map of mini-shell/CsoSCA-C showing the high resolution of the shell and the lower resolution of the internal density. Map gaussian filtered to two standard deviations in ChimeraX; left: density map at high contour (0.05); right: density map at low contour (0.01) and clipped to show internal features. Color representation of the local resolution is shown in the key. **d,** The asymmetric unit of mini-shell/CsoSCA-C, consisting of three CsoS1A monomers (cyan) and one CsoS4A monomer (magenta), is fitted to the density map.

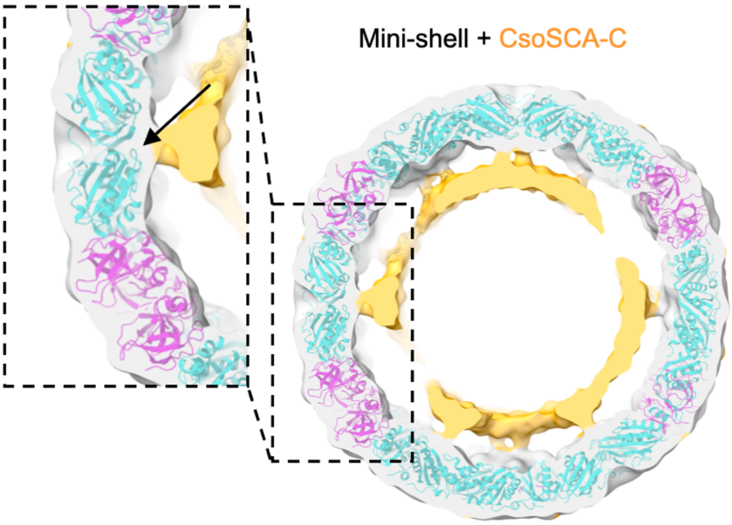

Supplementary Figure 12. Cryo-EM density map of purified mini-shell/CsoSCA-C. The map is shown at low contouring and fitted with the *T*=4 mini-shell model determined previously (PDB: 8B11). The map and atomic model are clipped at the front and back to allow clear visualization of the internal density. The mini-shell model is shown in cartoon representation (cyan for CsoS1A, magenta for CsoS4A) and the internal density on the map is coloured in yellow (map has been gaussian filtered and contoured at 0.01). Inset: the arrow indicates the connection between the internal density and the central pore of a CsoS1A hexamer. Conversely, no contacts are seen between the inner density and CsoS4A.

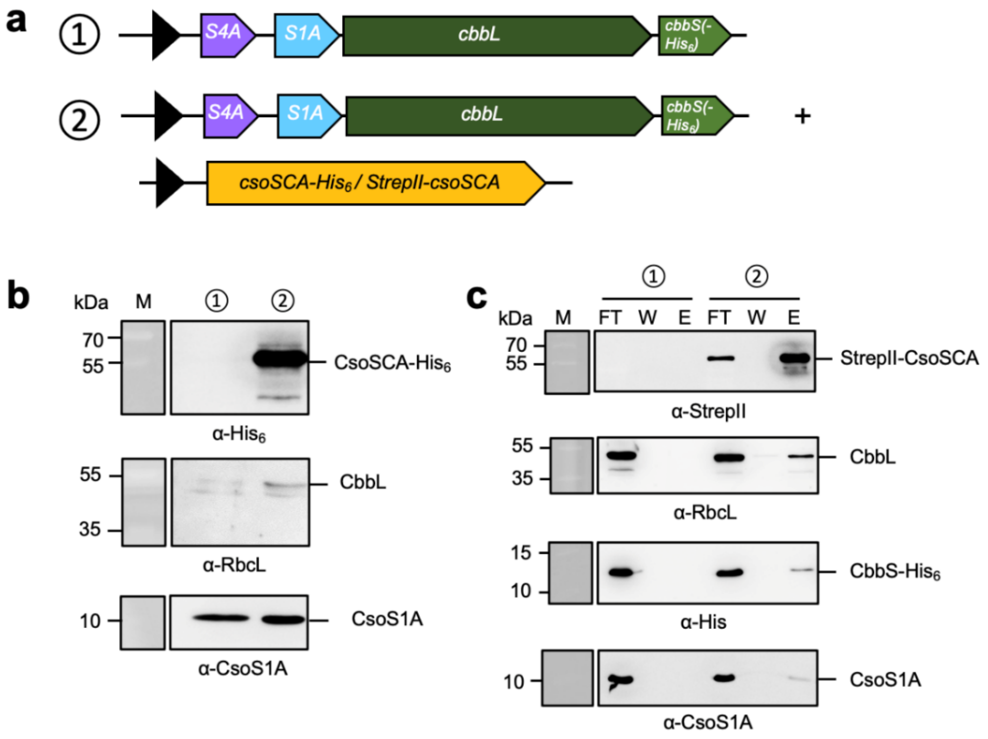

Supplementary Figure 13. CsoSCA interacts with Rubisco and CsoS1A. a, Schematic diagram of the experimental set-up. *H. neapolitanus* Rubisco (*cbbLS* in **b,** *cbbLS-His₆* in **c**) was cloned after the genes encoding for mini-shells on the same construct; the order of genes encoding for mini-shells and Rubisco are swapped on this construct compared to the *H. neapolitanus cso* operon to ensure high expression of mini-shell proteins. The mini-shell-Rubisco construct was co-expressed with full length *csoSCA*. Two differently tagged CsoSCA constructs were used; CsoSCA with a C-terminal 6xHis tag (same construct as shown in Fig. 3) was used in **b,** CsoSCA with an N-terminal Strep-tag II was used in **c. b,** Immunoblotting of purified mini-shells from the experiment described in **a**. Samples were normalized to the same amount of CsoS1A proteins detected by α-CsoS1A. **c,** *In vivo* pulldown assay of sample 1 and sample 2, using StrepII-CsoSCA as the bait protein. The flow-through (FT), final wash step (W) and elution (E) of the pulldown assay were analyzed by immunoblotting.

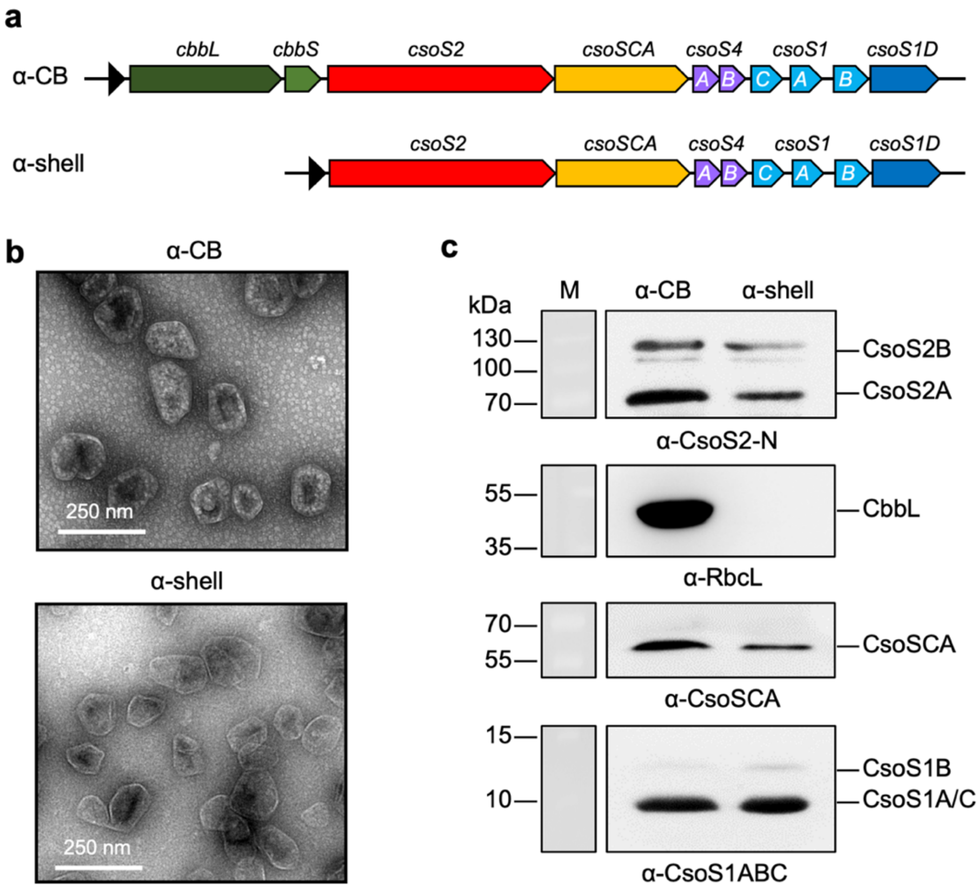

Supplementary Figure 14. CsoSCA is present in α-carboxysome shells lacking Rubisco. a, Schematic representation of the experiment. α-CB represents α-carboxysome and α-shell represents α-carboxysome shell. The α-CB construct contains all the genes from the *H. neapolitanus cso* operon, as well as *csoS1D*. The α-shell construct lacks the *cbbLS* genes in α-CB which encodes for Rubisco. **b,** Negative-stain transmission EM and **c,** Immunoblotting of purified α-carboxysomes/α-shells from the experiment described in **a**. In **c,** samples were normalized to the same amount of CsoS1A and CsoS1C proteins detected by α-CsoS1ABC.

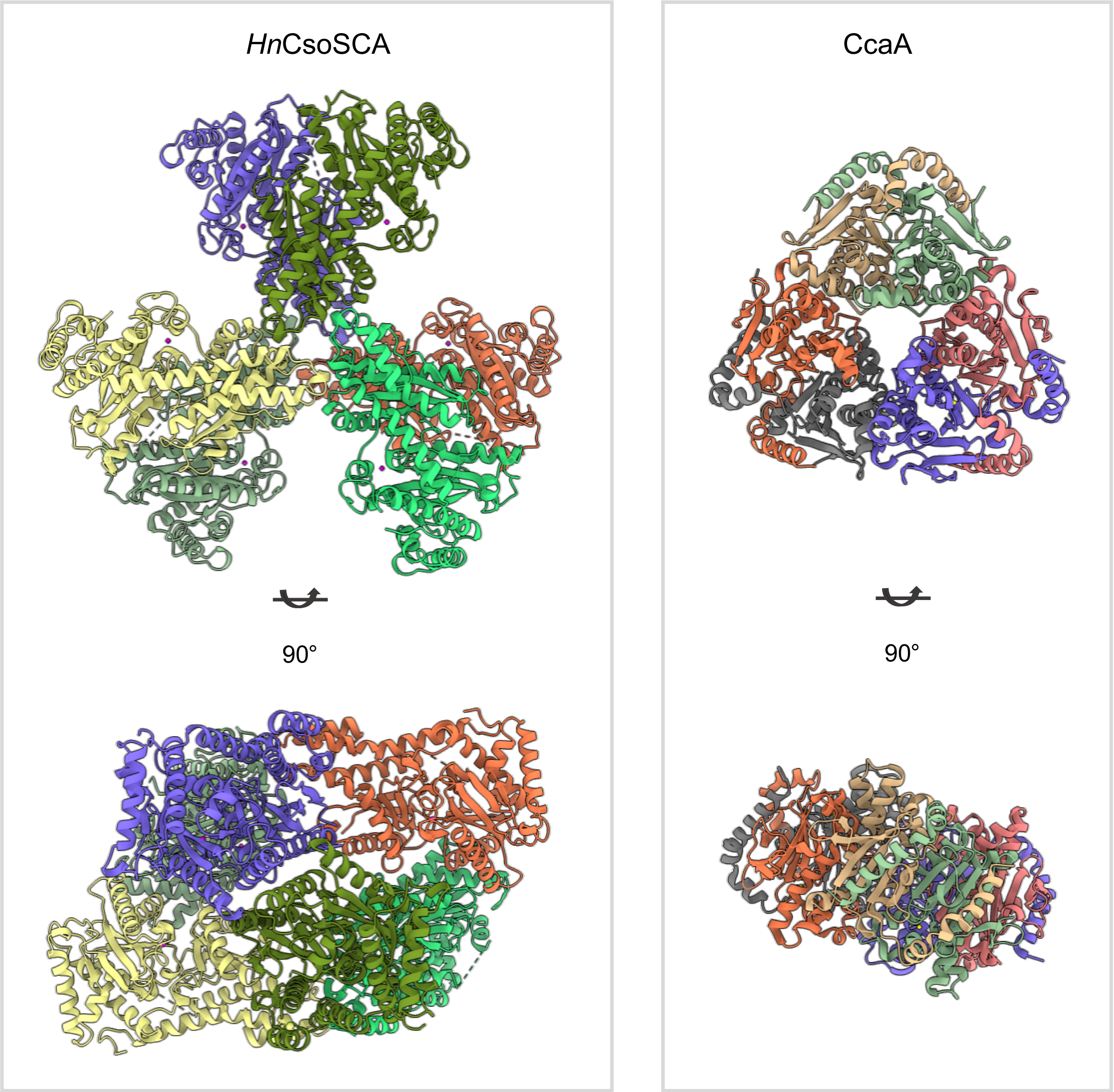

Supplementary Figure 15. Comparison of β-CA from α- and β-carboxysomes. Left: *Hn*CsoSCA, representing β-CA from α-carboxysomes. Right: CcaA from *Synechocystis* sp. PCC 6803 (PDB: 5SWC), representing β-CA from β-carboxysomes. Structures are shown in front and top views. Each protomer in the hexamer is represented by a distinct color. Zinc ions are depicted as pink (*Hn*CsoSCA) or yellow (CcaA) spheres.

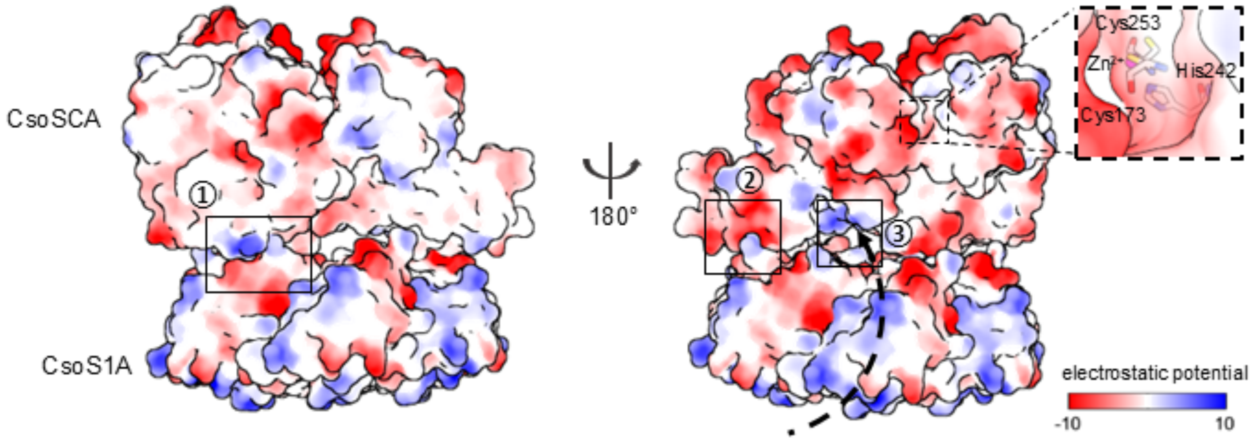

Supplementary Figure 16. Possible interactions between CsoSCA and CsoS1A. The electrostatic potential surfaces of CsoSCA and CsoS1A from Fig. 5a are shown. Only one CsoS1A hexamer and one CsoSCA monomer are shown for clarity. The Coulombic electrostatic potential was calculated and visualized on ChimeraX, with blue representing positive potential and red representing negative potential. The color scale of the electrostatic potential surface is in units of kcal/(mol·*e*) at 298 K. Interfaces showing electrostatic complementarity are highlighted in boxes 1 and 2. The arrow indicates the movement of HCO_3^- as it passes through the central pore of CsoS1A. The positively charged patch on CsoSCA that faces the central pore of CsoS1A is highlighted in box 3. The position of the active site of CsoSCA is highlighted on the structure by a dashed box, and the catalytic residues shown as sticks and labelled in the inset.

Supplementary Table 1. Cryo-EM data collection, refinement and validation statistics.

	***Hn*CsoSCA**	**Mini-shell/CsoSCA$^{\Delta C}$**	**Mini-shell/CsoSCA-C**
**Data collection**			
Microscope, magnification	Thermo Scientific Glacios, 240,000	Titan Krios, 105,000	Thermo Scientific Glacios, 240,000
Detector	Falcon IV	BioQuantum K3	Falcon IV
Voltage (kV)	200	300	200
Electron exposure (e^{-} Å^{-2})	50	50.49	50
Defocus range (μm)	-2.0 to -0.6	-2.0 to -0.6	-2.0 to -0.6
Pixel size (Å)	0.574	0.825	0.574
Micrographs collected	7,997	9,998	2,198
Total extracted particles (no.)	2,324,430	1,234,561	69,513
**Reconstruction**			
EM Data Bank	EMD-51067	EMD-51633	EMD-51641
PDB	9G4T	9GVC	9GW1
EMPIAR	EMPIAR-12910	EMPIAR-12896	EMPIAR-12950
Symmetry imposed	D3	I	I
Final particle images (no.)	435,444	47,086	3,003
Map resolution (Å) 0.143 FSC threshold	2.51	1.82	3.05
Map sharpening *B* factor (Å^2)	113	41	56
**Model refinement**			
Initial model used (PDB code)	2FGY	8B11	8B11
Model resolution (Å) 0.143/0.5 FSC threshold	2.47/2.62	1.81/1.89	2.90/3.20
Non-hydrogen atoms	3600	2635	2572
Protein residues	458	359	357
Ligands	1	0	0
B factors (Å^2)			
Protein	28.39	44.61	118.34
Ligand	57.91	-	-
R.m.s.d.			
Bond lengths (Å)	0.003	0.003	0.004
Bond angles (º)	0.515	0.503	0.566
Validation			
MolProbity score	1.88	1.03	1.42
Clashscore	5.61	2.47	7.65
Poor rotamers (%)	6	5	6
Ramachandran plot			
Favored (%)	98.24	99.43	98.85
Allowed (%)	1.76	0.57	1.15
Disallowed (%)	0.00	0.00	0.00

Supplementary Table 2. Primers used in this study.

Name	Sequence (5' to 3')	Purpose
NPC_oligo_4	ttttgttctacgtaagcttcgaattttaggcttgtggcgccttagg	Reverse primer for amplification of *csoS2-MC-4A-1A* and *csoS2-C-4A-1A* from pBAD::S2-4A-1A
NPC_oligo_19	gccaggatccgaattcgagct	Forward primer for amplification of pBAD33
NPC_oligo_20	catggtatatctccttcttgaattcgctagc	Reverse primer for amplification of pBAD33
NPC_oligo_23	tagcgaattcaagaaggagatataccatgaacacccgtaacacacgaagc	Forward primer to amplify *csoSCA*, *csoSCA-N* or *csoSCA*$^{\Delta C}$ for Gibson assembly with NPC_oligo_19+20 amplified pBAD33
NPC_oligo_24	gagctcgaattcggatcctggctcagtggtgatgatggtgatgtgcggatgca	Reverse primer to amplify *csoSCA* and introduce C-terminal 6x-His tag
NPC_oligo_26	gagctcgaattcggatcctggctcagtggtgatgatggtgatgtgc	Second reverse primer to amplify *csoSCA-His*$_6$ for Gibson assembly with NPC_oligo_19+20 amplified pBAD33
NPC_oligo_33	ttttgggctaacaggaggaattaaccatgaaggttgaaaccggtcacacc	Forward primer for amplification of *csoS2-MC-4A-1A* from pBAD::S2-4A-1A
NPC_oligo_44	tttgggctaacaggaggaattaaccatgtcaactgaacaatcattgacctgtg	Forward primer for amplification of *csoS2-C-4A-1A* from pBAD::S2-4A-1A
NPC_oligo_46	tcagtggtgatgatggtgatgtgcggatgcaacctcttcaatcggg	Reverse primer to amplify *csoSCA-C* and introduce C-terminal 6x-His tag
NPC_oligo_47	gagctcgaattcggatcctggctcagtggtgatgatggtgatgtgcggatgca	Second reverse primer to amplify *csoSCA-C-His*$_6$ for Gibson assembly with NPC_oligo_19+20 amplified pBAD33
NPC_oligo_48	tcagtggtgatgatggtgatgatgaccaatatcattgggtggatagcgacca	Reverse primer to amplify *csoSCA*$^{\Delta C}$ and introduce C-terminal 6xHis tag
NPC_oligo_49	gagctcgaattcggatcctggctcagtggtgatgatggtgatgatgaccaatatcattgg	Second reverse primer to amplify *csoSCA*$^{\Delta C}$*-His*$_6$ for Gibson assembly with NPC_oligo_19+20 amplified pBAD33
NPC_oligo_51	tcagtggtgatgatggtgatgcttctcagacattattttacgactgaattgttccgtgg	Reverse primer to amplify *csoSCA-N* and introduce C-terminal 6x-His tag
NPC_oligo_52	gctcgaattcggatcctggctcagtggtgatgatggtgatgcttctcaga	Second reverse primer to amplify *csoSCA-N-His*$_6$ for Gibson assembly with NPC_oligo_19+20 amplified pBAD33
NPC_oligo_53	agcgaattcaagaaggagatataccatggctgagcgatatatcagtgttggtgatggttt	Forward primer to amplify *csoSCA-C* for Gibson assembly with NPC_oligo_19+20 amplified pBAD33
NPC_oligo_54	tagggatccgaattcgagctcc	Forward primer for amplification of pETM11::TwinStrep-GB1-CsoSCA
NPC_oligo_61	agctcgaattcggatccctattatgcggatgcaacctcttcaatc	Reverse primer to amplify *csoSCA*-C for Gibson assembly with NPC_oligo_54+99 amplified pETM11::TwinStrep-GB1
NPC_oligo_62	agctcgaattcggatccctattacttctcagacattattttacgactgaattgttcc	Reverse primer to amplify *csoSCA-N* for Gibson assembly with NPC_oligo_54+99 amplified pETM11::TwinStrep-GB1
NPC_oligo_99	gccctgaaaatacaggttttcagtagtggggatgtcgtaatcgctc	Reverse primer for amplification of pETM11::TwinStrep-GB1-CsoSCA
NPC_oligo_100	tactgaaaacctgtattttcagggcaacac	Forward primer to amplify *csoSCA-N* or *csoSCA*$^{\Delta C}$

	ccgtaacacacgaagc	for Gibson assembly with NPC_oligo_54+99 amplified pETM11::TwinStrep-GB1
NPC_oligo_101	tactgaaaacctgtattttcagggcgctgag cgatatatcagtgttggt	Forward primer to amplify *csoSCA-C* for Gibson assembly with NPC_oligo_54+99 amplified pETM11::TwinStrep-GB1
NPC_oligo_111	aattcgaagcttacgtagaacaaaaactc atctcagaaga	Forward primer for amplification of pBAD::4A-1A. Fragment to be used for Gibson assembly with *cbbLS.*
NPC_oligo_112	catgagttcctcctgaaaggatgttaggctt gtggcgccttaggc	Reverse primer for amplification of pBAD::4A-1A. Fragment to be used for Gibson assembly with *cbbLS.*
NPC_oligo_113	catcctttcaggaggaactcatggcagtta aaaagtatagtgctggtg	Forward primer to amplify *cbbLS* for Gibson assembly with NPC_oligo_111+112 amplified pBAD::4A-1A
NPC_oligo_114	gtttttgttctacgtaagcttcgaattttagttgc cgcggtagacca	Reverse primer to amplify *cbbLS* for Gibson assembly with NPC_oligo_111+112 amplified pBAD::4A-1A
NPC_oligo_119	gagctcgaattcggatccctatcaatgacc aatatcattgggtggatagc	Reverse primer to amplify *csoSCA*$^{\Delta C}$ for Gibson assembly with NPC_oligo_54+99 amplified pETM11::TwinStrep-GB1
NPC_oligo_162	atcagcttggtcacatccacaatttgaaaa atataaactgattctgaacggcaaaaccct	Forward primer for amplification of pETM11::His$_6$-GB1-CsoSCA to exclude 6x-His and introduce N-terminal Twin-Strep tag. PCR amplification starts from *gb1* sequence
NPC_oligo_163	atgtgaccacatggtatatctccttcttaaag ttaaacaaaattatttctagag	Reverse primer for amplification of pETM11::His$_6$-GB1-CsoSCA to exclude 6x-His and introduce N-terminal Twin-Strep tag
NPC_oligo_164	aaggtggtggttcaggtggtggttcaggtg gttcatcagcttggtcacatccacaatttg	Second forward primer to introduce more of the N-terminal Twin-Strep tag sequence on the PCR product of NPC_oligo_162+163
NPC_oligo_165	actgggtctcaccacaatttgaaaaaggtg gtggttcaggtggtg	Forward primer to introduce BsaI site for Golden Gate cloning of pETM11::TwinStrep-GB1-CsoSCA
NPC_oligo_166	tgacggtctctgtggatgtgaccacatggta tatctccttcttaaagt	Reverse primer to introduce BsaI site for Golden Gate cloning of pETM11::TwinStrep-GB1-CsoSCA
NPC_oligo_168	actgggtctcaaaaaaacacccgtaacac acgaagcaagcaacgcgc	Forward primer for amplification of pETM11::TwinStrep-GB1-CsoSCA and introduce BsaI sites for Golden Gate cloning of pETM11::TwinStrep-CsoSCA
NPC_oligo_169	tgacggtctcatttttcaaattgtggatgtgac caagctgatgaaccacctgaaccacca	Forward primer for amplification of pETM11::TwinStrep-GB1-CsoSCA and introduce BsaI sites for Golden Gate cloning of pETM11::TwinStrep-CsoSCA
NPC_oligo_257	aactgccatggttaattcctcctgttagccca aaaaacgggt	Reverse primer for amplification of pBAD::α-shell
NPC_oligo_258	aagatgcgcatgaacaacattgatttgcgc gtctattcgtttatcga	Forward primer for amplification of pBAD::α-shell from the *csoS1D* sequence

NPC_oligo_259	taacaggaggaattaaccatggcagttaaaaagtatagtgctggtgtaaaagaataccgg	Forward primer to amplify *cbbLS-csoS2* for Gibson assembly with NPC_oligo_257+258 amplified pBAD and *csoSCA-4AB-1CAB*
NPC_oligo_260	cgggtgttcatgatcgttacactttacttaatcaaccgcgcgc	Reverse primer to amplify *cbbLS-csoS2* for Gibson assembly with NPC_oligo_257+258 amplified pBAD and *csoSCA-4AB-1CAB*
NPC_oligo_261	aaagtgtaacgatcatgaacacccgtaacacacgaagcaagc	Forward primer to amplify *csoSCA-csoS4AB-csoS1CAB* for Gibson assembly with *cbbLS-csoS2* and NPC_oligo_257+258 amplified pBAD
NPC_oligo_262	atcaatgttgttcatgcgcatcttccctactagacattagctattcagatttgc	Reverse primer to amplify *csoSCA-csoS4AB-csoS1CAB* for Gibson assembly with *cbbLS-csoS2* and NPC_oligo_257+258 amplified pBAD
NPC_oligo_292	aacacccgtaacacacgaagcaagca	Forward primer to amplify *csoSCA-His*$_6$ and the pBAD33 backbone
NPC_oligo_293	caaattgtggatgtgaccacatggtatatctccttcttgaattcgctagcccaaa	Reverse primer to amplify *csoSCA-His*$_6$ and the pBAD33 backbone and introduce the N-terminal Strep-tag II sequence
NPC_oligo_294	aaaggtggtagcggtggtagtaacacccgtaacacacgaagcaagca	Second forward primer to amplify the NPC_oligo_292+293 PCR product for Gibson assembly and introduce an N-terminal GS linker connecting *csoSCA-His*$_6$ and the Strep-tag II sequence
NPC_oligo_295	ccaccgctaccacctttttcaaattgtggatgtgaccacatggtatatctccttcttgaa	Second forward primer to amplify the NPC_oligo_292+293 PCR product for Gibson assembly and introduce an N-terminal GS linker connecting *csoSCA-His*$_6$ and the Strep-tag II sequence
NPC_oligo_296	tcgaattttagtggtgatgatggtgatggttgccgcggtagaccacgaag	Reverse primer to amplify pBAD::4A1A-CbbLS for Gibson assembly and introduce C-terminal 6x-His coding sequence to *cbbS*
NPC_oligo_297	caccatcatcaccactaaaattcgaagcttacgtagaacaaaaactcatctcagaagagg	Forward primer to amplify pBAD::4A1A-CbbLS for Gibson assembly and introduce C-terminal 6x-His coding sequence to *cbbS*
NPC_oligo_325	atccgcatgagccaggatccgaattcgagctc	Forward primer to amplify pBAD33 and *StrepII-csoSCA* for Gibson assembly
NPC_oligo_326	gatcctggctcatgcggatgcaacctcttcaatcggg	Reverse primer to amplify pBAD33 and *StrepII-csoSCA* for Gibson assembly

Supplementary Table 3. Vectors used in this study.

Name	Backbone	Description	Source
pHnCBS1D	-	Expression construct of the *Halothiobacillus neapolitanus cso* operon and CsoS1D	Addgene plasmid #52065
pBAD::α-CB	pBAD	pBAD derivative with entire *H. neapolitanus cso* operon (*cbbL* to *csoS1B*) and *csoS1D* cloned into PCR linearized backbone by Gibson assembly	This study
pBAD::α-shell	pBAD	pBAD derivative with *H. neapolitanus cso* operon from *csoS2* to *csoS1B* and *csoS1D* cloned into NcoI/EcoRI sites by Gibson assembly	(1)
pBAD::4A-1A	pBAD	pBAD derivative with *H. neapolitanus csoS4A* and *csoS1A* cloned into NcoI/EcoRI sites by Gibson assembly	(2)
pBAD::S2-4A-1A	pBAD	pBAD derivative with *H. neapolitanus csoS2, csoS4A* and *csoS1A* cloned into NcoI/EcoRI sites by Gibson assembly	(2)
pBAD::S2-MC-4A-1A	pBAD	pBAD derivative with *H. neapolitanus csoS2* M- and C-terminal regions (Lys264-Gly873), *csoS4A* and *csoS1A* cloned into NcoI/EcoRI sites by Gibson assembly. The *csoS2-MC-4A-1A* insert was amplified from pBAD::S2-4A-1A	This study
pBAD::S2-C-4A-1A	pBAD	pBAD derivative with *H. neapolitanus csoS2* C-terminal region (Ser608-Gly873), *csoS4A* and *csoS1A* cloned into NcoI/EcoRI sites by Gibson assembly. The *csoS2-C-4A-1A* insert was amplified from pBAD::S2-4A-1A	This study
pBAD::4A-1A-CbbL-CbbS	pBAD	pBAD derivative with *H. neapolitanus csoS4A, csoS1A* and *cbbLS* cloned into PCR linearized backbone by Gibson assembly. The pBAD backbone and the *csoS4A-csoS1A* sequence were amplified from pBAD::4A-1A and assembled with PCR amplified *cbbLS*	This study
pBAD::4A-1A-CbbL-CbbS-His_6	pBAD	pBAD derivative containing *H. neapolitanus csoS4A, csoS1A* and *cbbL* and *cbbS-*His_6. The entire sequence of pBAD::4A-1A-CbbL-CbbS was PCR amplified and the C-terminal 6x-His tag fused to CbbS by including the coding sequence on PCR primers. The plasmid was assembled by Gibson assembly of the linear PCR product	This study
pBAD33::CsoSCA-His_6	pBAD33	pBAD33 derivative with *H. neapolitanus csoSCA* fused to a C-terminal 6x-His tag, cloned into PCR linearized backbone by Gibson assembly	This study
pBAD33::StrepII-CsoSCA-His_6	pBAD33	pBAD33 derivative with *H. neapolitanus csoSCA* fused to an N-terminal Strep-tag II sequence as well as a C-terminal 6x-His tag. The entire sequence of	This study

		pBAD33::CsoSCA-His$_6$ was PCR amplified and N-terminal Strep-tag II sequence introduced by including the coding sequence on PCR primers. The plasmid was assembled by Gibson assembly of the linear PCR product	
pBAD33::StrepII-CsoSCA	pBAD33	pBAD33 derivative with *H. neapolitanus csoSCA* fused to an N-terminal Strep-tag II sequence.<br>The pBAD33 backbone and the *StrepII-csoSCA* sequence was amplified from pBAD33::StrepII-CsoSCA-His$_6$, and the linear PCR product assembled by Gibson assembly	This study
pBAD33::CsoSCA-N-His$_6$	pBAD33	pBAD33 derivative with *H. neapolitanus csoSCA-N* (Met1-Lys150) fused to a C-terminal 6x-His tag cloned into PCR linearized backbone by Gibson assembly	This study
pBAD33::CsoSCA$^{\Delta C}$-His$_6$	pBAD33	pBAD33 derivative with *H. neapolitanus csoSCA*$^{\Delta C}$ (Met1-His397) fused to a C-terminal 6x-His tag cloned into PCR linearized backbone by Gibson assembly	This study
pBAD33::CsoSCA-C-His$_6$	pBAD33	pBAD33 derivative with *H. neapolitanus csoSCA-C* (Ala398-Ala514) fused to a C-terminal 6x-His tag cloned into PCR linearized backbone by Gibson assembly	This study
pETM11::TwinStrep-GB1-CsoSCA	pETM11	pETM11 derivative with N-terminal TwinStrep-GB1-TEV sequence fused to *H. neapolitanus csoSCA.* The *pETM11::gb1-TEV-csoSCA* sequence was amplified from pETM11::His$_6$-GB1-CsoSCA (not used in this study), the N-terminal Twin-Strep fusion introduced by PCR, and the plasmid circularized by BsaI Golden Gate cloning	This study
pETM11::TwinStrep-CsoSCA	pETM11	pETM11::TwinStrep-GB1-CsoSCA derivative. Inverse PCR of the template plasmid followed by BsaI Golden Gate assembly generated *H. neapolitanus csoSCA* fused only to a N-terminal Twin-Strep tag	This study
pETM11::TwinStrep-GB1-CsoSCA-N	pETM11	pETM11 derivative with N-terminal TwinStrep-GB1-TEV sequence fused to *H. neapolitanus csoSCA-N* (Met1-Lys150). The *pETM11::TwinStrep-gb1-TEV* sequence was amplified from pETM11::TwinStrep-GB1-CsoSCA as the linear backbone and assembled with the *csoSCA-N* insert by Gibson assembly	This study
pETM11::TwinStrep-GB1-CsoSCA$^{\Delta C}$	pETM11	pETM11 derivative with N-terminal TwinStrep-GB1-TEV sequence fused to *H. neapolitanus csoSCA*$^{\Delta C}$ (Asn2-His397). The *pETM11::TwinStrep-gb1-TEV* sequence was amplified from pETM11::TwinStrep-	This study

		GB1-CsoSCA as the linear backbone and assembled with the *csoSCA*$^{\Delta C}$ insert by Gibson asssembly	
pETM11::TwinStrep-GB1-CsoSCA-C	pETM11	pETM11 derivative with N-terminal TwinStrep-GB1-TEV sequence fused to *H. neapolitanus csoSCA-C* (Ala398-Ala514). The *pETM11::TwinStrep-gb1-TEV* sequence was amplified from pETM11::TwinStrep-GB1-CsoSCA as the linear backbone and assembled with the *csoSCA-C* insert by Gibson asssembly	This study

Supplementary References

1. T. Li *et al.*, Reprogramming bacterial protein organelles as a nanoreactor for hydrogen production. *Nature Communications* **11**, 1-10 (2020).
2. T. Ni *et al.*, Intrinsically disordered CsoS2 acts as a general molecular thread for α-carboxysome shell assembly. *Nature Communications* **14**, 5512 (2023).
